# Comparative Genomic Analyses of the Human *NPHP1* Locus Reveal Complex Genomic Architecture and Its Regional Evolution in Primates

**DOI:** 10.1371/journal.pgen.1005686

**Published:** 2015-12-07

**Authors:** Bo Yuan, Pengfei Liu, Aditya Gupta, Christine R. Beck, Anusha Tejomurtula, Ian M. Campbell, Tomasz Gambin, Alexandra D. Simmons, Marjorie A. Withers, R. Alan Harris, Jeffrey Rogers, David C. Schwartz, James R. Lupski

**Affiliations:** 1 Department of Molecular and Human Genetics, Baylor College of Medicine, Houston, Texas, United States of America; 2 Laboratory for Molecular and Computational Genomics, Department of Chemistry, Laboratory of Genetics and The UW-Biotechnology Center, University of Wisconsin-Madison, Madison, Wisconsin, United States of America; 3 Graduate Program in Diagnostic Genetics, School of Health Professions, University of Texas MD Anderson Cancer Center, Houston, Texas, United States of America; 4 Human Genome Sequencing Center, Baylor College of Medicine, Houston, Texas, United States of America; 5 Department of Pediatrics, Baylor College of Medicine, Houston, Texas, United States of America; 6 Texas Children’s Hospital, Houston, Texas, United States of America; University of Washington, UNITED STATES

## Abstract

Many loci in the human genome harbor complex genomic structures that can result in susceptibility to genomic rearrangements leading to various genomic disorders. Nephronophthisis 1 (NPHP1, MIM# 256100) is an autosomal recessive disorder that can be caused by defects of *NPHP1*; the gene maps within the human 2q13 region where low copy repeats (LCRs) are abundant. Loss of function of *NPHP1* is responsible for approximately 85% of the NPHP1 cases—about 80% of such individuals carry a large recurrent homozygous *NPHP1* deletion that occurs via nonallelic homologous recombination (NAHR) between two flanking directly oriented ~45 kb LCRs. Published data revealed a non-pathogenic inversion polymorphism involving the *NPHP1* gene flanked by two inverted ~358 kb LCRs. Using optical mapping and array-comparative genomic hybridization, we identified three potential novel structural variant (SV) haplotypes at the *NPHP1* locus that may protect a haploid genome from the *NPHP1* deletion. Inter-species comparative genomic analyses among primate genomes revealed massive genomic changes during evolution. The aggregated data suggest that dynamic genomic rearrangements occurred historically within the *NPHP1* locus and generated SV haplotypes observed in the human population today, which may confer differential susceptibility to genomic instability and the *NPHP1* deletion within a personal genome. Our study documents diverse SV haplotypes at a complex LCR-laden human genomic region. Comparative analyses provide a model for how this complex region arose during primate evolution, and studies among humans suggest that intra-species polymorphism may potentially modulate an individual’s susceptibility to acquiring disease-associated alleles.

## Author Summary

Genomic instability due to the intrinsic sequence architecture of the genome, such as low copy repeats (LCRs), is a major contributor to *de novo* mutations that can occur in the process of human genome evolution. LCRs can mediate genomic rearrangements associated with genomic disorders by acting as substrates for nonallelic homologous recombination. Juvenile-onset nephronophthisis 1 is the most frequent genetic cause of renal failure in children. An LCR-mediated, homozygous common recurrent deletion encompassing *NPHP1* is found in the majority of affected subjects, while heterozygous deletion representing the nephronophthisis 1 recessive carrier state is frequently observed amongst world populations. Interestingly, the human *NPHP1* locus is located proximal to the head-to-head fusion site of two ancestral chromosomes that occurred in the great apes, which resulted in a reduction of chromosome number from 48 in nonhuman primates to the current 46 in humans. In this study, we characterized and provided evidence for the diverse genomic architecture at the *NPHP1* locus and potential structural variant haplotypes in the human population. Furthermore, our analyses of primate genomes shed light on the massive changes of genomic architecture at the human *NPHP1* locus and delineated a model for the emergence of the LCRs during primate evolution.

## Introduction

Genomic instability is a major contributor to *de novo* mutations that can occur in the process of human genome evolution [1–3]. Genomic rearrangements can be mediated by various mechanisms, including nonallelic homologous recombination (NAHR), nonhomologous end joining, mobile element insertion (e.g. long interspersed element (LINE)-mediated retrotransposition) and replication based mechanisms [4]. Low copy repeat (LCR) mediated NAHR plays a significant role in genomic instability resulting in rearrangements associated with genomic disorders [5]. LCRs, also known as segmental duplications, are two or more repeated sequences that usually span 10–400 kilobases (kb) each and share >95% DNA sequence identity [6,7]. LCRs are highly homologous, and constitute ~5–10% of the human and great ape genomes [6,8,9]. LCRs provide substrates for NAHR-mediated crossing-over that results in structural variants (SVs) including copy number variants (CNVs) such as duplications and deletions of large genomic segments [5] or copy number neutral events such as inversions [10–12].

Numerous NAHR-mediated rearrangements are associated with genomic disorders by affecting dosage sensitive genes. For example, Potocki-Lupski syndrome (PTLS, MIM #610883) or Smith-Magenis syndrome (SMS, MIM #182290) are frequently caused by an ~3.7 megabases (Mb) NAHR-mediated common recurrent duplication or deletion, respectively. These recurrent rearrangements of 17p11.2 utilize directly oriented proximal and distal SMS-REPs as substrates for NAHR [13–18].

LCRs originated from genomic evolutionary processes and can facilitate responses to selective pressure by creating new genes that may contribute to lineage-specific phenotypes. LCRs can also configure local genomic structure in a manner that contributes significantly to disease susceptibility [19–24]. Because of their repetitive nature and structural complexity, LCRs can confound the accuracy of human and nonhuman mammalian genome assemblies. Discerning long stretches of paralogous, highly identical sequences can be difficult; this problem becomes particularly challenging when there are more than two copies in a haploid genome [6,25,26], and consequently LCRs are likely under-represented in draft genome assemblies for many species. Mappability of the short sequencing reads from next generation sequencing techniques can be reduced within LCRs, and as a result multiple experimental molecular and computational approaches are often required to characterize SVs relative to the human haploid reference in a given personal genome. Several efforts have demonstrated the value of thoroughly scrutinizing complex genomic regions to better understand the human genome and discern variation that may be important to health, evolution, and susceptibility to diseases [27–33].

The human chromosomal region 2q13-2q14.1 represents the product of head-to-head fusion of two ancestral chromosomes forming human chromosome 2 [34]. This evolutionary fusion event is unique to the human genome, and is responsible for the chromosome number difference (46 versus 48) between human and the great apes including chimpanzee (*Pan troglodytes*), gorilla (*Gorilla gorilla*) and orangutan (*Pongo abelii*). The fusion of two subtelomeric regions from two ancestral chromosomes (analogous chromosomes 2A and 2B in the great apes) introduced substantial complexity to this region. A common recurrent 290 kb deletion encompassing Nephrocystin-1 (*NPHP1*, MIM *607100), a gene that maps to the centromeric portion of the human 2q13 region, is associated with several diseases. Juvenile-onset nephronophthisis 1 (NPHP1, MIM #256100) is an autosomal recessive cystic kidney disorder causing chronic renal failure in children. Homozygous *NPHP1* deletion is found in ~80% of patients born to consanguineous parents and in ~60% of sporadic cases [35]. In addition to nephronophthisis 1, the same *NPHP1* deletion has also been identified in patients with Senior-Loken syndrome-1 (SLSN1, MIM# 266900) and Joubert syndrome 4 (JBTS4, MIM# 609583) with distinct phenotypes [35–37]. Moreover, a recent study demonstrates that heterozygous *NPHP1* deletion CNV in combination with *NPHP1* point mutations (SNVs) can lead to Bardet-Biedl syndrome (BBS, MIM# 209900) [38]. The *NPHP1* deletion is recurrent, and results from NAHR-mediated unequal crossing-over involving the directly oriented flanking LCRs [39]; the frequency of heterozygous *NPHP1* deletion is estimated to be approximately 1/400 in normal individuals from northern European descent [38]. Dittwald *et al* explored a clinical database containing chromosomal microarray (CMA) data from 25,144 patients, of which *NPHP1* duplications (N = 233) and deletions (N = 118) were found to be the most commonly observed copy number aberrations (combined ~1.4%) compared to CNVs from other loci [5].

The complex genomic architecture of the human 2q13 region, especially the *NPHP1* locus, provides the foundation for different SVs that may be observed in personal genomes among human populations. The polymorphic nature of this locus was previously demonstrated, and its evolution, prevalence and potential impact to disease susceptibility warrant further investigation. In this study, we utilized a combination of genomic technologies, including array-comparative genomic hybridization (aCGH) and optical mapping (OM), to identify novel SV haplotypes at the *NPHP1* locus and clarify the relative frequencies of specific haplotypes in the human population. We further utilized comparative sequence alignments of primate genome sequences and aCGH to construct a model for the evolution of the genomic architecture at the *NPHP1* locus in nonhuman primates and human genomes. Unexpectedly, we found that this region displays evidence for incomplete lineage sorting, such that the structure of this region in humans is more similar to that of gorillas than to the orthologous region in chimpanzees or orangutans. The results also confirmed a dramatic genomic expansion of the *NPHP1* locus during primate evolution and revealed a pattern of LCR evolution that may be explained by a model of multi-step, serial segmental duplication [32].

## Results

### Computational characterization of the *NPHP1* locus in the human reference genome

**Fig 1:**
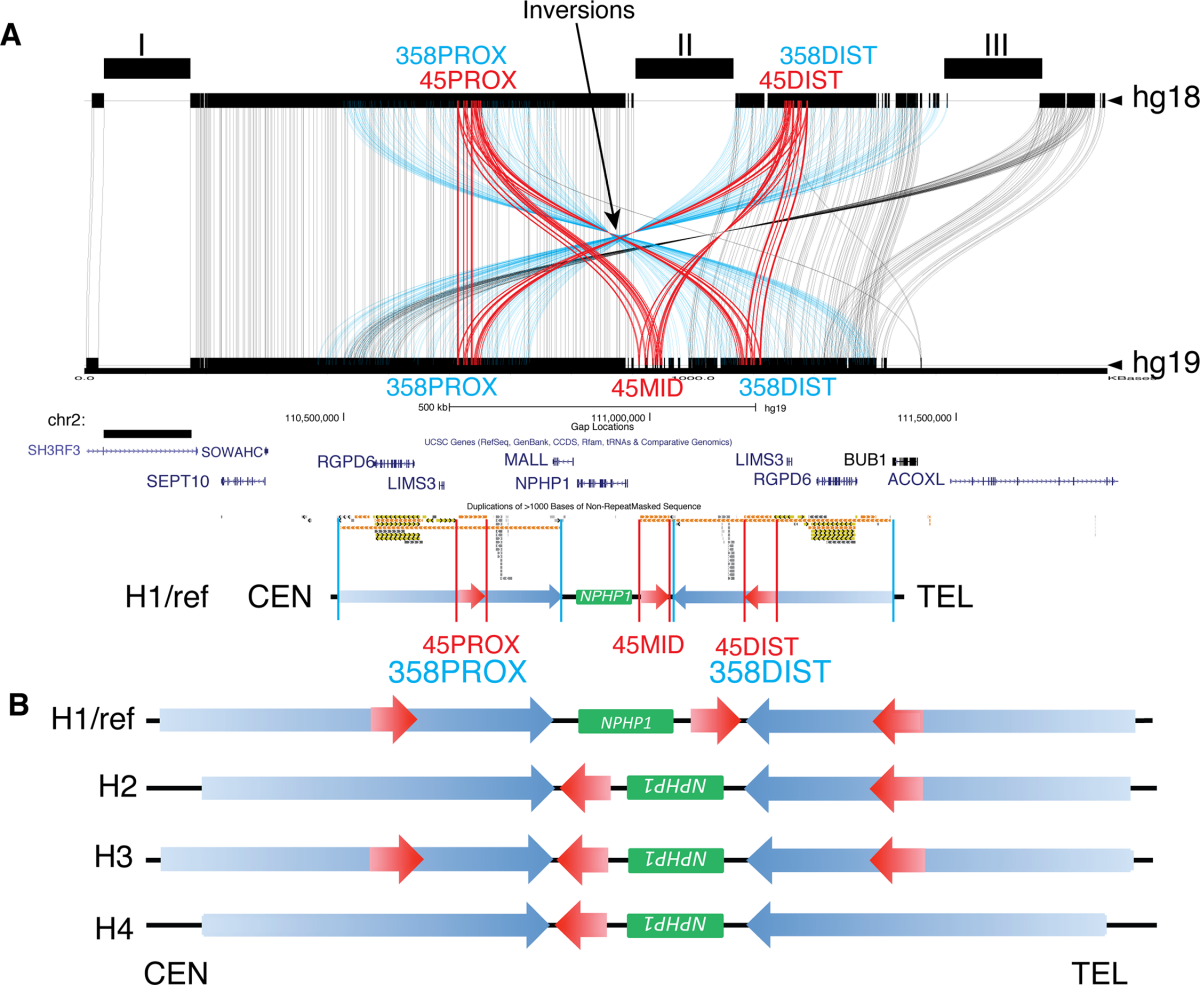
Known SV haplotypes at the *NPHP1* locus in the human 2q13 region. **A.** Comparison between human reference builds hg18 and hg19 by Miropeats. The traces between the top and bottom track represent the aligned paralogous sequences greater than 1 kb in size between the two builds. Inverted orientations of paralogous LCRs could be inferred (e.g. 45PROX and 45DIST, 358PROX and 358DIST) using the intersection point in a cluster as the indicator. Blue traces, aligned sequences from the 358 kb LCRs; red traces, aligned sequences from the 45 kb LCRs. Top track, human reference build hg18; bottom track, human reference build hg19. The positions of gaps in build hg18 are shown above the top track. “Segmental Dups” track from UCSC Genome Browser is shown below the Miropeats plots with the diagram of the SV haplotypes of the reference build hg19 shown underneath. **B.** Known SV haplotypes at the *NPHP1* locus. Green block, *NPHP1* gene; blue arrows, the 358 kb LCRs; red arrows, the 45 kb LCRs; CEN, centromeric side (proximal); TEL, telomeric side (distal).

The exceedingly complex and polymorphic genomic architecture of the *NPHP1* locus presented difficulty during the assembly of the haploid human genome reference. This becomes readily apparent by the comparison between two recent updates of the human reference assembly, GRCh37/hg19 and NCBI36/hg18, at the *NPHP1* locus. The 800 kb sequences flanking each end of *NPHP1* in hg18 and hg19 were compared by Miropeats [40]. Two (Gaps II and III) of the three major gaps (Gaps I, II and III) in hg18 are closed in hg19; Gap II corresponds to a region encompassing an ~45 kb LCR distal to *NPHP1* that is only present in hg19 (**Fig 1A**).

We delineated the LCR structure of the *NPHP1* locus (hg19, chr2: 110,080,914–111,762,639) using data from the UCSC Genome Browser “Segmental Dups” track (http://genome.ucsc.edu/index.html). Two LCRs of approximately 358 kb in length, which we termed 358PROX (centromeric) and 358DIST (telomeric), flanked *NPHP1* in an inverted orientation–this can also be manifested by the Miropeats (**Fig 1A**). Similarly, two LCRs of approximately 45 kb in length, termed 45PROX (centromeric) and 45DIST (telomeric), were embedded within 358PROX and 358DIST, and thus had an inverted orientation. One additional LCR paralogous to 45PROX and 45DIST, termed 45MID, was revealed in build hg19 after the closure of Gap II from hg18, resulting in a total of three copies of the 45 kb LCRs in the haploid reference. The 45MID and 45PROX, flanking *NPHP1* in direct orientation, are presumably the LCR pairs responsible for the NAHR-mediated *NPHP1* deletion (**Fig 1A**). Self-alignments of the DNA sequences (chr2: 110,080,914–111,762,639) using NCBI BLAST tool (http://blast.ncbi.nlm.nih.gov/Blast.cgi) confirmed the genomic structure of the *NPHP1* locus in the reference by revealing the two major groups of LCRs and their relative orientations (**S1A Fig**).

We computationally characterized the LCRs at the *NPHP1* locus using the human reference sequences. Pairwise alignments of both groups of 45 kb LCRs and 358 kb LCRs against their individual consensus sequences revealed a high percentage of sequence identities for both the 358 kb LCRs and 45 kb LCRs (**Table 1**). PR domain-containing protein 9 (PRDM9) recognizes a degenerative 13-mer motif (5’-CCNCCNTNNCCNC-3’) that is critical to recruit recombination machinery required for crossovers in at least 40% of all human homologous recombination hot spots [4,41–44]. NAHR crossover studies suggest that the frequency of the PRDM9 hotspot motifs within LCR regions is one of the parameters correlated with the rate of NAHR mediated genomic rearrangement [5,17]. Characterization of the PRDM9 hot spot motif in the LCRs of the *NPHP1* locus may elucidate potential crossover sites. At the *NPHP1* locus, 12 motifs were found in each of the three 45 kb LCRs, while 161 and 154 hotspot motifs were found in 358PROX and 358DIST, respectively (**Table 1**). LCRs contribute to the complex genomic architecture of this region, and could incite genomic instability. The high degree of sequence identity between LCR pairs and the density of PRDM9 hotspot motifs (**Table 1, S1B Fig**) may additionally contribute to the instability and increase the recurrent rearrangement frequency at the *NPHP1* locus. The high similarity (>99.6%) between the corresponding paralogous LCRs in humans also indicates that gene conversion may occur frequently at the *NPHP1* region [45].

**Table 1:** Features of the LCRs at the human *NPHP1* locus.

LCR1	Chr	Start	Stop	Number of NAHR hotspot motifs	LCR2	Chr	Start	Stop	Number of NAHR hotspot motifs	Relative orientation between LCR1 and LCR2	Fraction matching (%)	Pairwise alignment (with internal gap penalty)
45PROX	2	110688765	110733137	12	45MID	2	110983704	111031088	12	+	99.7723	99.6644
45MID	2	110983704	111031088	12	45DIST	2	111153516	111197896	12	-	99.7836	99.7252
45DIST	2	111153516	111197896	12	45PROX	2	110688765	110733137	12	-	99.8805	99.7860
358PROX	2	110494431	110852754	161	358DIST	2	111033787	111392192	154	-	99.8841	99.7170

### Structural polymorphisms at the human *NPHP1* locus

NAHR events between directly oriented LCRs generate deletions or duplications; while NAHR events between inverted-oriented LCRs lead to inversions–such copy number neutral SVs may impose weaker selection forces than deletions and duplications do, and are thus more likely to be found as population polymorphisms [27–29]. In fact, the SV haplotype at the *NPHP1* locus identified in the reference genome, arbitrarily designated as the H1 SV haplotype, is not the only SV haplotype in the human population. Experimental evidence suggested the presence of at least three alternative SV haplotypes (H2, H3 and H4, **Fig 1**) before the first draft of the human genome assembly [39]. These alternative SV haplotypes share an inversion of the *NPHP1* region, encompassing *NPHP1* and 45MID, between 358PROX and 358DIST (*NPHP1* inversion). Besides the *NPHP1* inversion, H2 and H4 appear to have several other SVs unique to themselves – 45PROX was lost in H2, while both 45PROX and 45DIST were lost in H4 (**Fig 1B**).

**Fig 2:**
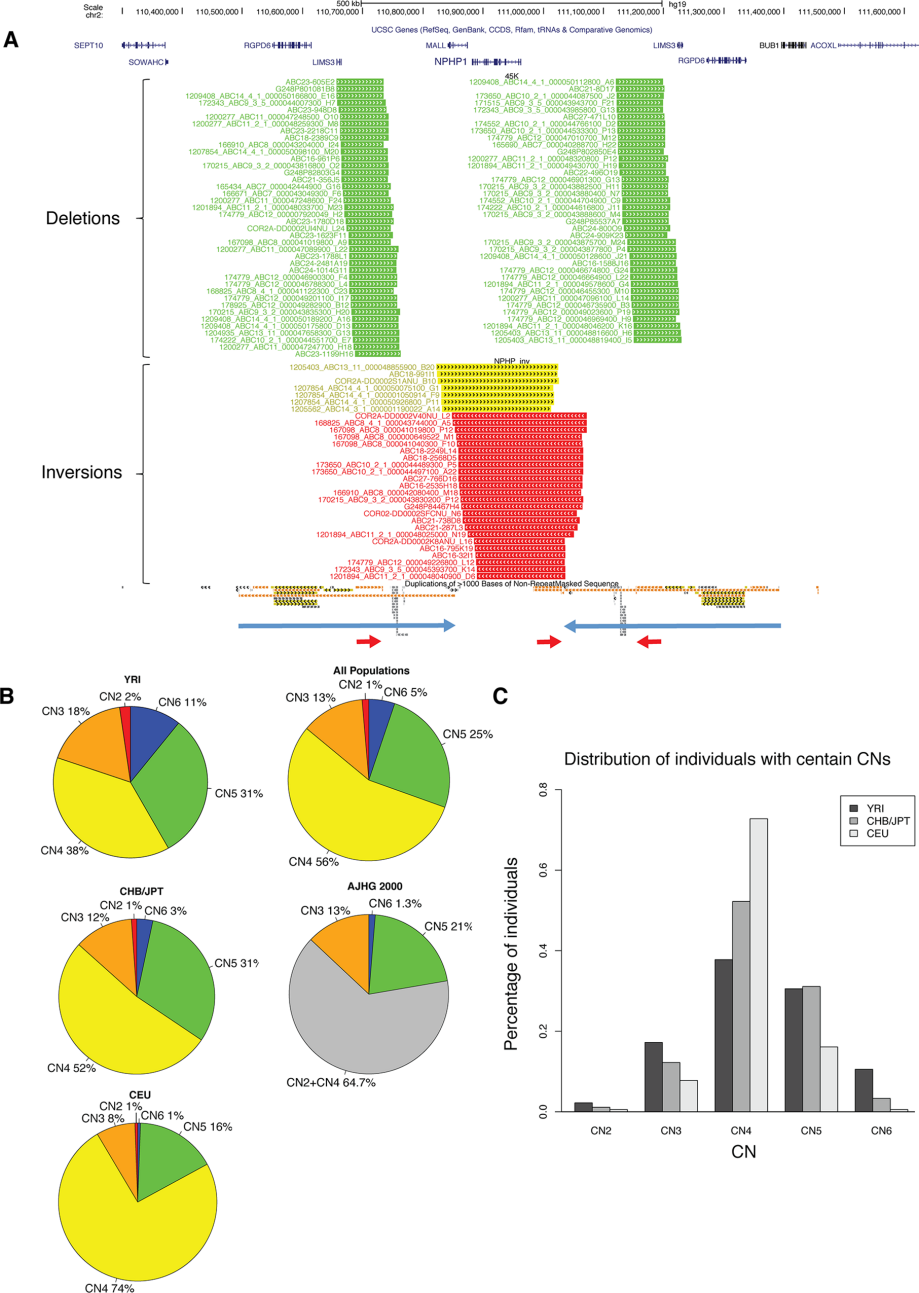
Copy number polymorphisms of the 45 kb LCRs. **A.** Discordant fosmids selected from individual fosmid libraries shown as custom track in UCSC Genome Browser. Diagram of SV haplotype of human reference is shown underneath the custom track. Green segments, discordant fosmids representing deletions; yellow segments, discordant fosmids representing inversions with one ESP mapped to the 358PROX; red segments, discordant fosmids representing inversions with one ESP mapped to the 358DIST. **B.** Pie chart showing copy number distribution of the 45 kb LCRs in each population. Percentage of each copy number (CN) is plotted. CHB and JPT are combined. YRI, Yoruba in Ibadan, Nigeria; CHB, Han Chinese in Beijing, China; JPT, Japanese in Tokyo, Japan; CEU, CEPH (Utah residents with ancestry from northern and western Europe); all populations, an aggregate of all the populations investigated in Conrad *et al*. 2010; AJHG 2000, population data in Saunier *et al*. 2000 [39]; Red, CN2 in 2% of YRI, 1% of CHB/JPT and 1% of CEU; orange, CN3 in 18% of YRI, 12% of CHB/JPT and 8% of CEU; yellow, CN4 in 38% of YRI, 52% of CHB/JPT and 74% of CEU; green, CN5 in 31% of YRI, 31% of CHB/JPT and 16% of CEU; blue, CN6 in 11% of YRI, 3% of CHB/JPT and 1% of CEU; grey, CN2 plus CN4 (the frequencies of which were not specifically indicated by Saunier *et al*. 2000). **C.** Bar plot showing copy number distribution of the 45 kb LCRs in each population. Percentage of each copy number is plotted.

We hypothesized that the majority of the structural polymorphism at the *NPHP1* locus can derive from (1) the *NPHP1* inversion, (2) the copy number loss of one or more of the 45 kb LCRs, or (3) a combination of these two events. To obtain further evidence to support these SV haplotypes and their prevalence across human populations, we examined the fosmid libraries from the Human Genome Structural Variation project (HGSV, http://humanparalogy.gs.washington.edu/structuralvariation/) to search for individual discordant fosmids representing SVs [12]. We identified 78 discordant fosmids representing losses of either 45PROX or 45DIST and 31 discordant clones representing *NPHP1* inversions between 358PROX and 358DIST in a total number of 17 individuals (**Fig 2A**). Each of the 17 individuals had at least one discordant fosmid indicating loss of the 45 kb LCR, while 13/17 had at least one discordant fosmid indicating the *NPHP1* inversion, suggesting that the current human reference genome actually presents a minor SV allele. We further examined the copy number distribution of the 45 kb LCRs utilizing the dataset published by Conrad *et al* [2]. CNVs in 450 individuals from different ethnicity groups were genotyped. These individuals include 180 CEU (Utah residents with ancestry from northern and western Europe), 180 YRI (Yoruba in Ibadan, Nigeria), 45 JPT (Japanese in Tokyo, Japan) and 45 CHB (Han Chinese in Beijing, China). Various copy numbers of the 45 kb LCRs, ranging from two to six, were observed at different frequencies in each population (**Fig 2B**). The distributions of copy numbers across different populations were not significantly different (Kruskal-Wallis rank sum test, p-value = 0.6766, **Fig 2C**). Aggregating all the populations, 1%, 13%, 56%, 25% and 5% of the entire examined population has two, three, four, five and six copies of the 45 kb LCRs, respectively (**Fig 2B**). The frequencies of copy number derived from Conrad *et al* also correlated well with those derived from PFGE experimental data by Saunier *et al* [39], in which 13%, 21% and 1.3% of 152 control individuals from an undefined ethnicity were found to harbor three, five and six copies of 45 kb LCRs, respectively (**Fig 2B**). It is likely that the most common copy number of the 45 kb LCRs in a diploid genome is four, which deviates from the copy number of six that would exist in an individual with homozygous H1, as in the haploid reference genome. Thus the six-copy state may be a minor genotype that was represented in only 1.3%-5% of the general population.

### Potential novel SVs revealed by a combination of OM and aCGH approaches

**Fig 3:**
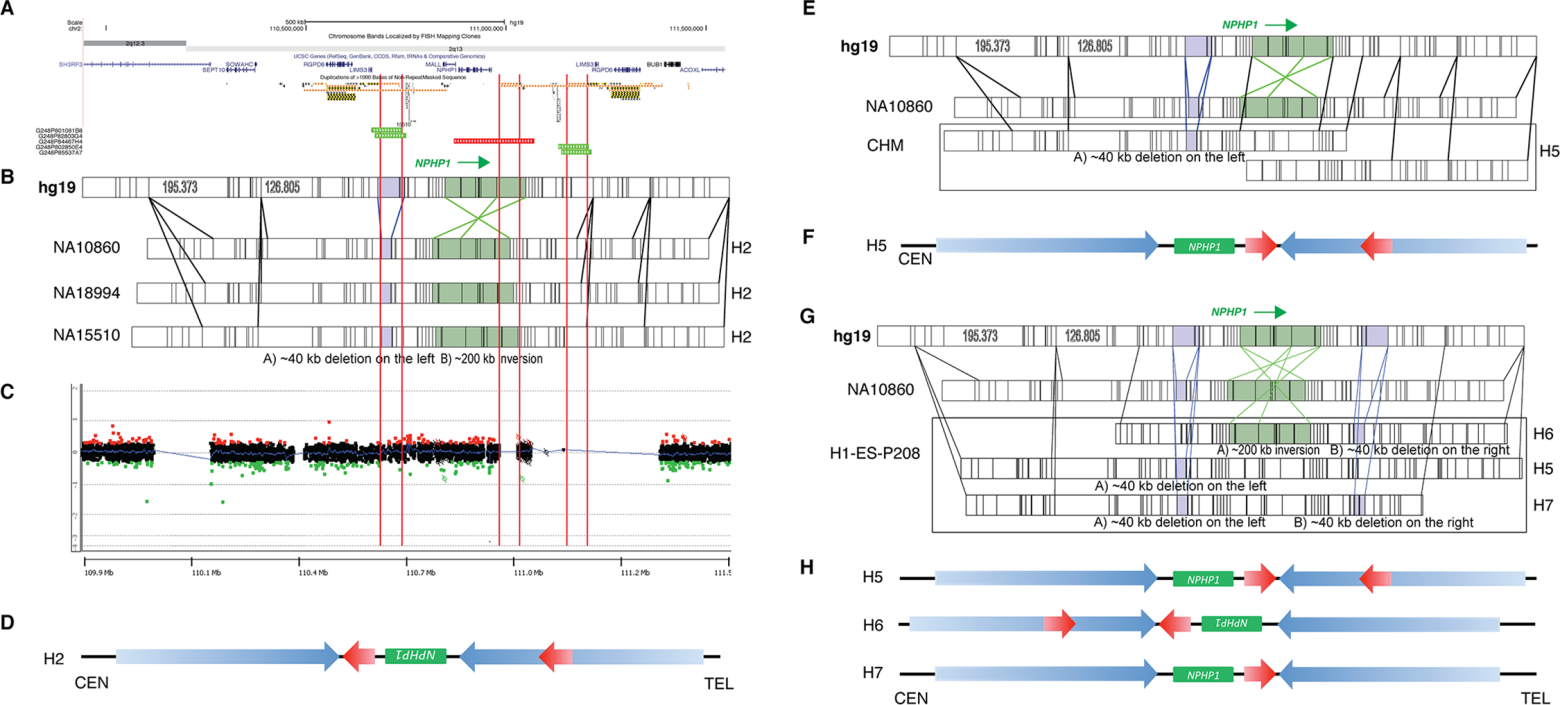
SV haplotypes at the *NPHP1* locus delineated by OM and aCGH. **A.** UCSC Genome Browser custom track showing discordance fosmids identified in NA15510. Discordant fosmids representing *NPHP1* inversion (red) and 45 kb LCR deletion (green) are shown on the custom track. **B.** SV haplotypes delineated by OM analysis. Four OM diagrams, from top to bottom, represent the *in silico* Rmap of hg19, the SwaI Rmap of NA10860, NA18994 and NA15510, respectively. Vertical black lines, individual restriction sites; purple area, the 45 kb LCR and its deletion product; green area, *NPHP1* region and its inversion product. **C.** aCGH result of NA15510. The log_2_ ratio plot is shown. No CNV can be observed from the plot. **D.** Diagram showing the H2 SV haplotype of NA15510. The diagram results from data obtained from both OM and aCGH. **E.** OM results of CHM. **F.** Diagram showing the SV haplotype of CHM. Only one consensus map is found, which supports the H5 SV haplotype. **G.** OM results of H1-ES-P208. **H.** Diagram showing the SV haplotypes of H1-ES-P208. In total, three consensus maps are identified, indicating three different SV haplotypes (H5, H6 and H7).

The observation of polymorphic structural variants, including copy number polymorphisms of the 45 kb LCRs and *NPHP1* inversion, prompted us to search for novel SV haplotypes. OM constructs ordered restriction maps (Rmaps) from single-molecules of DNA, which are assembled into genome-wide contigs that can be compared to an *in silico* restriction map from the human reference in order to discern SVs [46]. The OM can be used as an independent validation method for SVs revealed by other methods, such as fosmid sequencing data, which suggested SVs including the *NPHP1* inversion and loss of the 45 kb LCRs in HapMap individual NA15510 (**Fig 3A**). The SwaI OM contig assembly of NA15510 and its alignment to an *in silico* human reference (hg19) further validated a homozygous H2 SV haplotype at the interrogated locus (chr2: 109,943,987–111,547,676), with four copies of the 45 kb LCRs and homozygous *NPHP1* inversion (**Fig 3B**). Additionally, NA10860 and NA18994 yielded OM results supporting genotypes identical to NA15510 (**Fig 3B**).

Fluorescence *in situ* hybridization (FISH) was performed in an attempt to delineate the organization of *NPHP1* and the 45 kb LCRs at the *NPHP1* locus. FISH experiments were designed with fosmid probes independently targeting *NPHP1* (fluoresces green, G) and the 45 kb LCRs (fluoresces red, R). A fosmid probe targeting ~1.3 Mb proximal to *NPHP1* was used as an “anchor probe” (fluoresces blue, B, **S2A Fig**). In the majority of interphase cells from the lymphoblastoid cell line of NA15510 (42/50), we observed resolved signals of four red and two green (R4G2), which represented four copies of the 45 kb LCR and two copies of *NPHP1* in a diploid genome (**S2B Fig**). This result was consistent with the copy number data from OM. Additionally, a signal pattern of red-green-red, representing an SV haplotype also consistent with the OM data, was observed in the interphase cells (**S2B Fig**); however, such organization of signals was confounded by a signal pattern of yellow-red, the yellow of which was likely to represent an overlapping signal between red and green due to a two dimensional representation of a three dimensional reality and the close physical proximity, or overlapping in the z–plane, of a red and green signal. Moreover, using the blue “anchor probe” as a third color, we observed a signal pattern of blue-red-green-red in 25/50 interphase cells examined. This experimental result was also consistent with the OM data observed for this sample (**S2C Fig**). However, such a pattern was not uniformly and consistently found (**S2C Fig**). In aggregate, these results may be explained by the close proximity of the components being targeted, the three dimensional relative spatial positioning, and the less organized structure of chromosomes in interphase cells.

We then performed custom-designed aCGH (**S3A Fig**) to validate the copy number estimations from OM. We used the DNA sample from NA10851, a genome that has four copies of the 45 kb LCRs, as the universal reference for aCGH experiments. As a proof of principle, aCGH comparing six DNA samples (NA18517, NA15510, NA18994, NA10860, NA18555 and NA12878) with NA10851 confirmed the copy number of the 45 kb LCRs estimated by the Conrad *et al* study (**Fig 3C, S3B Fig**). These samples included the three samples (NA15510, NA18994, NA10860) interrogated by OM. The consistency of copy numbers predicted by Conrad *et al*, aCGH, OM and the corroboration of the independent experimental assays including FISH further substantiated the accuracy of OM analysis for discerning CNV/SVs.

A total number of eight DNA samples were analyzed by OM, and the contig assemblies at the *NPHP1* locus revealed different SV haplotypes (**Table 2**). In addition to NA10860/NA18994/NA15510, MM52 (a multiple myeloma primary tumor sample) [47] and HF087 (an oligodendroglioma primary sample) [48] were also found to be homozygous for the H2 SV haplotype by OM (**Table 2**). In the DNA from CHM (complete hydatidiform mole, CHM1h-TERT) [46], the OM consensus map showed an allele with the loss of the 45PROX but without the *NPHP1* inversion, presenting a potential novel SV haplotype (termed H5, **Fig 3E and 3F, Table 2**). CHM is derived from fertilization of an enucleated egg with a single sperm [29], and the haploid nature of the CHM genome facilitates accurate mapping and assembly by eliminating allelic variations from the diploid genomes. The OM analysis of H1-ES-P208 (human embryonic stem cell line, passage 208) revealed alleles with three different SV haplotypes, including one H5 SV haplotype and two other novel SV haplotypes, one with the loss of the 45DIST and the *NPHP1* inversion (termed H6) and the other one with losses of both 45PROX and 45DIST without the *NPHP1* inversion (termed H7) (**Fig 3G and 3H, Table 2**). It is interesting that three different SV haplotypes in H1-ES-P208 were identified by OM, suggesting potential mosaicism. An NAHR-mediated inversion could potentially occur between the inverted 358 kb LCRs to convert H5 to H6 or *vice versa*. The highly identical paralogous LCRs may facilitate this rearrangement during the additional mitoses from the 208 cell culture passages of H1-ES-P208 [49,50]. aCGH analysis of H1-ES-P208 supported the copy number of three in its diploid genome as inferred by the OM analysis. Thus it led to the possibility that an admixture of cells with H5/H7 or H6/H7 combinations, both of which represent three copies of the 45 kb LCRs, could be present in the H1-ES-P208 cell line (**Table 2**). This finding should be further validated using orthogonal approaches that may delineate SV haplotypes. Unfortunately, the H1-ES-P208 cell line is no longer available. The DNA sample from HCC1937 (a lymphoblastoid cell line from primary ductal carcinoma) revealed two different SV haplotypes, H2 and H5. These results emphasize the structural complexity of the *NPHP1* locus and indicate that two (or potentially sometimes more reflecting mosaic states) SV haplotypes may be observed in the genome of one individual. However, although novel SV haplotypes are identified based on data from the aforementioned cell lines, it remains to be examined how representative they are of the different human populations worldwide.

**Table 2:** Structural haplotype delineation by combined OM and aCGH approach.

Sample name	OM		aCGH	Potential haplotype
	Number of allele types	Description	45 kb LCR CN^5^	
NA10860	1	45PROX loss, *NPHP1* inversion	4	H2
NA18994	1	45PROX loss, *NPHP1* inversion	4	H2
NA15510	1	45PROX loss, *NPHP1* inversion	4	H2
H1-ES-P208^1^	3	(1) 45PROX loss; (2) 45DIST loss, *NPHP1* inversion; (3) 45PROX loss, 45DIST loss	3	H5, H6 and H7
MM52	1	45PROX loss, *NPHP1* inversion	NA	H2 (without aCGH)
HF087^2^	1	45PROX loss, *NPHP1* inversion	NA	H2 (without aCGH)
CHM^3^	1	45PROX loss	NA	H5 (without aCGH)
HCC1937^4^	2	(1) 45PROX loss, *NPHP1* inversion; (2) 45PROX loss	NA	H2 and H5 (without aCGH)

^1^ H1 ES cell line–Passage 208

^2^ Oligodendroglioma primary sample [48]

^3^ Complete hydatidiform mole (CHM1h-TERT), haploid genome

^4^ Lymphoblastoid cell line from primary ductal carcinoma

^5^ Copy number

### Comparative analysis reflecting evolution of the 45 kb LCRs

The 45 kb LCRs are responsible for the NAHR-mediated recurrent *NPHP1* deletion. We have shown above that the copy number of this LCR is highly dynamic in the human population. To better understand the origin of this complexity and assess homologous genomic regions in closely related species, we investigated this region in great apes (chimpanzees, gorillas and orangutans) and Old World monkeys (rhesus macaques [*Macaca mulatta*] and baboons [*Papio anubis*]). Analysis of the evolutionary history of this interval may represent a unique opportunity to characterize the emergence of a repeat sequence that causes susceptibility to a specific disease in humans. We were able to trace the human 45 kb LCR locus back to its ancestral origin by comparing several nonhuman primate genomes with the SV haplotypes observed in humans.

**Fig 4:**
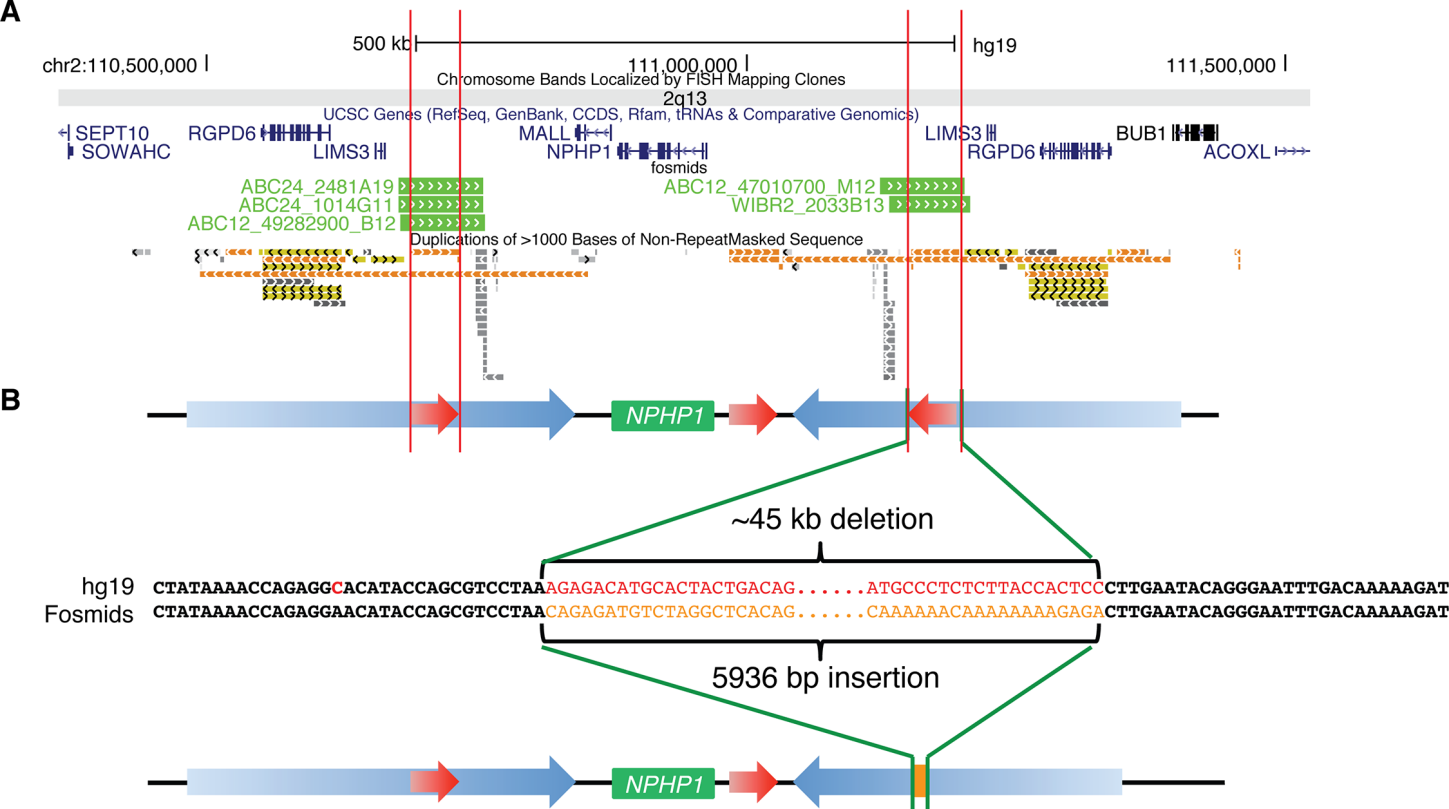
Deletion of the 45 kb LCRs is accompanied with a 5936 bp insertion (5936Ins). **A.** UCSC Genome Browser custom track of the five fosmids (WIBR2-2033B13, ABC12-49282900B12, ABC12-47010700M12, ABC24-1014G11 and ABC24-2481A19) with fully sequenced inserts. **B.** Diagram on the top shows the SV haplotype in the haploid reference. A hypothetical SV haplotype with 45DIST deleted from the reference is shown at the bottom. This hypothetical SV haplotype is shown only for the purpose of illustrating the deletion/insertion haplotype. In the middle, the breakpoints of the 45 kb LCR deletion and 5936Ins are shown at base pair resolution. The full insert sequence of the fosmid from individual ABC12 can be found in **S3 Fig.**

Based on the OM data, the deletion of the 45 kb LCR resulted in an ~40 kb loss in the human subjects compared to the human reference, with an ~6 kb mismatching sequence remaining at the place of the 45 kb LCR loss (**Fig 3B**). Alignments of human discordant fosmid clones with fully sequenced inserts, mapped to either the proximal or the distal side of human *NPHP1* (hg19), revealed a shared “deletion/insertion” haplotype with the 45 kb LCR deletion and a 5936 bp insertion (5936Ins) at the deletion breakpoint junction (**Figs 4A and 3B, S4 Fig**). The 5936 bp stuffer sequence could not be uniquely mapped to any position of the human genome reference hg19 using BLAT (http://genome.ucsc.edu/cgi-bin/hgBlat). We further investigated the origin of the 5936Ins using BLAST (http://blast.ncbi.nlm.nih.gov/Blast.cgi) with the Nucleotide collection (nr/nt) database. Interestingly, in addition to the five fully sequenced clones previously found in the human fosmid libraries, we found two chimpanzee BAC clones, CH251-328D3 and CH251-71L9, which encompassed a sequence highly identical (98%) to the human 5936Ins. The two clones mapped to a region in the chimpanzee chromosome 2A –a position syntenic to the human *NPHP1* locus. This finding indicated that the 5936Ins that was present at some but not all human *NPHP1* loci was also present in at least some chimpanzee genomes. We subsequently used BLAT to search for this sequence in the genomes of baboon, rhesus macaque, orangutan, gorilla and chimpanzee. This 5936Ins could be aligned to the reference genomes of all these species with increasing sequence identities, meaning it may exist as an ortholog in Old World monkeys and great apes (**Table 3**).

**Table 3:** BLAT result of the 5936Ins and the human 45MID in non-human primates.

Species	Features	Templates		Identity	Individual reference genomes			Span	Positions relative to *NPHP1*	Reference genome
		Start	End		Chr	Start	End			
Baboon	5936Ins	6	5924	92.7%	13	101017341	101022874	5534	Proximal	Mar. 2012 (Baylor Panu_2.0/papAnu2)
	45 kb LCR	-	-	-	-	-	-	-	-	Same as above
Macaque	5936Ins	1	5924	92.9%	13	109439800	109445316	5517	Proximal	Oct. 2010 (BGI CR_1.0/rheMac3)
	45 kb LCR	-	-	-	-	-	-	-	-	Same as above
Orangutan	5936Ins	1	5924	95.5%	2a	18250677	18256233	5557	Proximal	July 2007 (WUGSC 2.0.2/ponAbe2)
	45 kb LCR	9253	29725	96.3%	2a	18526564	18548764	22201	Middle	Same as above
Chimpanzee	5936Ins	6	5936	98.8%	2A	110402874	110408810	5937	Proximal	Feb. 2011 (CSAC 2.1.4/panTro4)
	45 kb LCR	4	40495	99.0%	2A	110634309	110676396	42088	Middle	Same as above
Gorilla	5936Ins	6	2670	98.3%	2A	107770907	107773574	2668	Proximal	May 2011 (gorGor3.1/gorGor3)
		4654	5936	97.3%	2A	107817505	107818162	658	Proximal	Same as above
		1	2714	98.4%	2A	108190039	108193136	3098	Distal	Same as above
	45 kb LCR	20767	25178	98.3%	2A	107791461	107795879	4419	Proximal	Same as above
		7519	45429	98.4%	2A	108148382	108183474	35093	Distal	Same as above

### Dynamic changes of genomic architecture surrounding the *NPHP1* locus during primate evolution

From ~85 million years ago (Mya) to the present, primate genomes have undergone substantial sequence change during evolution. Genomic segmental duplications have been one significant aspect of that process [21,51,52]. The human 5936Ins that is conserved among baboons, rhesus macaques and great apes motivated us to use comparative genomic analyses to understand the dynamic structural changes that occurred at the *NPHP1* locus during the evolution of the human genome.

**Fig 5:**
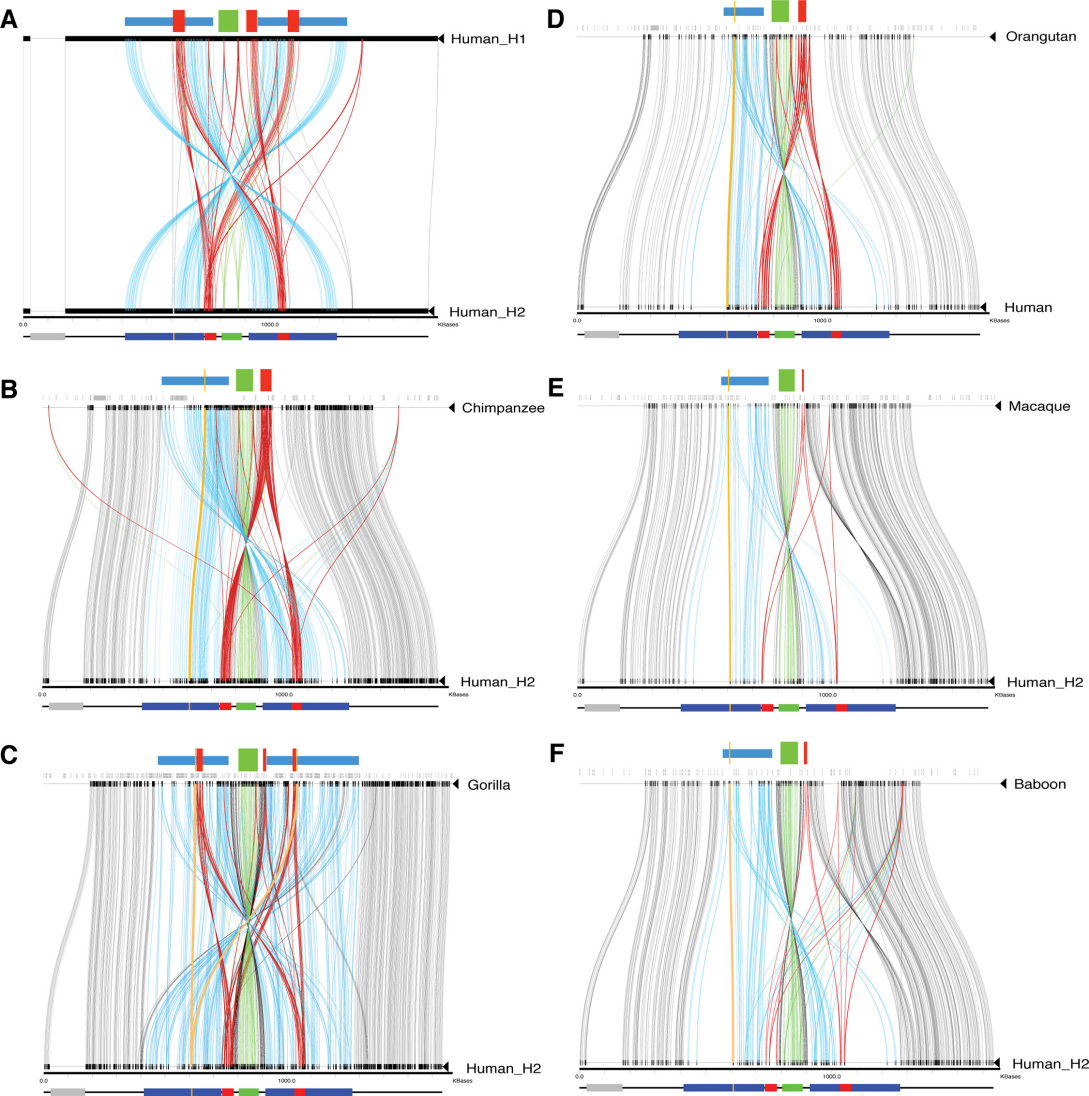
Inter-species Miropeats alignments of primates to the human H2 SV haplotype. **A.** Human reference build hg19 (H1). **B.** Chimpanzee (panTro4). **C.** Gorilla (gorGor3). **D.** Orangutan (ponAbe2). **E.** Macaque (rheMac3). **F.** Baboon (papAnu2). Above the top track in each Miropeats diagram is a depiction of the distribution of gaps (in grey) in the reference of nonhuman primates and a diagram of the genomic structure revealed by Miropeats. Below the bottom track in each Miropeats diagram is the diagram of H2 SV haplotype. Blue boxes, the human 358 kb LCRs or their orthologs in nonhuman primates; red boxes, the human 45 kb LCRs or their orthologs in nonhuman primates; yellow boxes, the human 5936Ins or its orthologs in nonhuman primates; green boxes, *NPHP1* in each primate; grey boxes, gaps in the references. Blue traces, aligned sequences between the human 358 kb LCRs and their nonhuman orthologs; red traces, aligned sequences between the human 45 kb LCRs and their nonhuman orthologs; yellow traces, aligned sequences between the human 5936Ins and its nonhuman orthologs.

DNA sequences of 800 kb flanking each side of *NPHP1* were downloaded from UCSC Genome Browser (http://genome.ucsc.edu/index.html) for baboon, rhesus macaque, orangutan, gorilla, chimpanzee and human. According to our previous analysis, a minor allele is presented at the *NPHP1* locus in the hg19. As a result, we modified the human reference sequence so that it reflected the more common H2 SV haplotype (**Table 2**). We manually constructed the H2 SV haplotype sequences by: (1) deleting the sequences of 45PROX, (2) inserting the 5936Ins and (3) inverting the sequences between 358PROX and 358DIST (**Fig 5A–5F**). Miropeats [40] was subsequently used to perform local alignments between the human H2 and each individual primate reference genome. Alignment between human H1 and H2 revealed the gain of the 45PROX in H1, the overall sequence similarity, and the inverted orientation of the paralogous 358 kb LCRs (**Fig 5A**). Old World monkeys, including baboons and rhesus macaques, diverged from the ancestors of humans about 25–33 million years ago (Mya) and share 94.9% sequence identity with the human genome [53]. Due to lower sequence identity between baboon/macaque and human, the Miropeats threshold was lowered to display the alignments between more distant species; this change in the threshold correlated with more noise in the alignments. The diagram of alignments suggested that sequences homologous to the human 45 kb LCRs do not exist in the current baboon/macaque reference genomes, as the only traces between these references and human H2 were sparse, and likely indicated noisy alignments (**Fig 5E and 5F**). A region on the left side of both baboon and rhesus macaque *NPHP1* could be aligned to a portion of both human 358 kb LCRs, indicating that a smaller region orthologous to part of the human 358 kb LCRs exists in both of the baboon and macaque references, but it appears as only a single copy per haploid genome (**Fig 5E and 5F**). In both baboon and rhesus macaque, the intra-species Miropeats alignments also revealed a lack of the pattern of paralogous LCRs, which was observed in the human reference genome (**S5E and S5F Fig**). The great ape lineages including orangutans, gorillas and chimpanzees diverged from the human evolutionary lineage about 12–16 Mya, 6–8 Mya and 4.5–6 Mya, respectively, with increasing sequence identity to the human genome (**S1 Table**) [53]. Miropeats revealed similar patterns of alignment of orangutan and chimpanzee’s references versus human H2: two paralogous 45 kb LCRs in the human reference were either directly or invertedly aligned to a single genomic region in the references of orangutan and chimpanzee; while the two 358 kb LCRs in the human reference were partially aligned to a single genomic region on the left side of *NPHP1* in the orangutan and chimpanzee references, with chimpanzee appearing to have a larger partial alignment (**Fig 5B and 5D**). Although the 45 kb and 358 kb LCRs in human could be aligned to a region in orangutan and chimpanzee references with longer sequence homology, these genomic segments were lacking paralogous LCR partners. Consistently, intra-species alignments by Miropeats did not reveal any pattern of paralogous LCRs in their reference genomes (**S5B and S5D Fig**).

Gorilla is a more distant species from human than chimpanzee according to the standard evolutionary phylogeny; however, it had a Miropeats pattern more consistent with that of a human-human alignment. Each of the two human 45 kb LCRs in H2 could be apparently aligned to three genomic regions surrounding *NPHP1* in the gorilla reference, suggesting an SV haplotype in gorilla that is orthologous but with lower sequence homology (**Fig 5C**). Moreover, each of the two human 358 kb LCRs could also be aligned to the flanking sequences of gorilla *NPHP1* at the paralogous positions, suggestive of a potential orthologous locus in gorilla (**Fig 5C**). Intra-species alignment of the gorilla reference genome to itself revealed paralogous alignment of long sequences flanking *NPHP1*, suggesting that a genomic architecture similar to the one observed in humans at the *NPHP1* locus at least in the gorilla reference (**S5C Fig**).

Miropeats also revealed the conservation of the human 5936Ins in the nonhuman primate genomes. As shown by Miropeats, the 5936Ins observed in the human H2 was also identified in the reference genomes of baboon, rhesus macaque, orangutan and chimpanzee **(Fig 5B, 5D–5F**). Interestingly, the gorilla reference contained two regions similar to the human 5936Ins that were adjacent to two gorilla 45 kb LCR orthologs (**Fig 5C**). The relative transitions of the human 45 kb LCRs and 5936Ins in different nonhuman primates derived from Miropeats were confirmed by BLAT using the sequences of the human 45MID and the 5936Ins as templates (**Table 3**). Since Miropeats was applied to reference genomes to reveal similarities and differences between humans and nonhuman primates, the comparison results may not represent the general populations of queried species. Orthogonal experimental approaches, including aCGH and copy number analysis of genomic sequencing data generated from multiple primate individuals, were used to further investigate the copy number changes in the nonhuman primates comparing to humans.

Interphase FISH experiments were performed on lymphoblastoid cell lines of one chimpanzee (CRL-1868) and one gorilla (CRL-1854) using the human fosmid probes described above to explore the genomic architecture at the *NPHP1* locus in each species. The experiment illustrated the 45 kb LCR orthologs in both species tested. The majority of the scored gorilla interphase cells (46/50) and all of the scored chimpanzee interphase cells (50/50) showed R2G2, suggesting one copy of the 45 kb LCR ortholog and one copy of *NPHP1* on the haploid genome of each individual (**S2D and S2E Fig**). Interestingly, resolved signals of R4G2 was observed in a minority population (4/50) of gorilla interphase cells, indicating a potential two-copy configuration of the 45 kb LCR ortholog in the gorilla haploid genome.

Inter-species aCGH were performed to further validate the copy number alterations indicated by sequence alignments (**S6A Fig**). Genomic DNAs from baboon (N = 1), rhesus macaque (N = 2), orangutan (N = 1), gorilla (N = 3) and chimpanzee (N = 7) were used to compare with human genomic DNA (NA10851) on the previously described aCGH. The quality of the hybridization positively correlated with the sequence identities between different primates and human (**S1 Table**). Comparing to the human genome, a large portion of genomic sequences flanking *NPHP1* appeared to be nonexistent or have lower copy number in nonhuman primates, and the degree of similarity to human varied from baboon to chimpanzee (**S6 Fig**). After careful examination of the aCGH data of the 358 kb LCR locus, we achieved an estimation of variation of the genomic content between nonhuman primates and human. Sequences totaling sizes of 146 kb (40.85%), 156 kb (43.6%), 88 kb (24.6%), 24 kb (6.7%) and 72 kb (20.1%) appeared to be nonexistent (aCGH log_2_ ratio was lower than -1) in the genomes of baboon, rhesus macaque, orangutan, gorilla and chimpanzee, respectively, while approximately 123 kb (34.4%), 93 kb (26.0%), 115 kb (32.1%), 156 kb (43.6%) and 197 kb (55.0%) appeared to exist in the genomes of these primates, albeit at lower copy number (aCGH log_2_ ratio was between 0 and -1). The latter observation indicated that these genomic regions might constitute the ancestral nonduplicated segments orthologous to the human LCRs. The indication of copy number variants is derived from the log_2_ ratio of aCGH probes, which largely relies on the degree of hybridization based on sequence similarity. Thus, these data can reflect genome differences and phylogenic distance between the two species being compared. The sizes of genomic segments with copy number changes described above are estimates, and may be refined by testing a large cohort of nonhuman primates.

Since the 45 kb LCRs in human are directly related to the recurrent *NPHP1* deletion, and a haplotype consisting of a 45 kb LCR loss accompanied with a 5936Ins is frequently observed, we were interested in understanding the evolution of this haplotype. As previously shown, the copy number of the 45 kb LCRs ranged from two to six in humans without *NPHP1* deletion. Moreover, aCGH of eight DNA samples from patients with homozygous recurrent *NPHP1* deletion revealed two copies of the 45 kb LCRs in seven individuals and four copies in one individual (**Table 4**). NAHR-mediated *NPHP1* deletion between a pair of directly-oriented 45 kb LCRs reduces one copy, creating a recombinant copy from two substrate copies, of the 45 kb LCRs after the deletion event. Thus the majority (14/16) of haplotypes examined prior to *NPHP1* deletion would have two copies of the 45 kb LCRs–this is consistent with the observation that copy number of two is the most frequently observed copy number in a haploid human genome.

**Fig 6:**
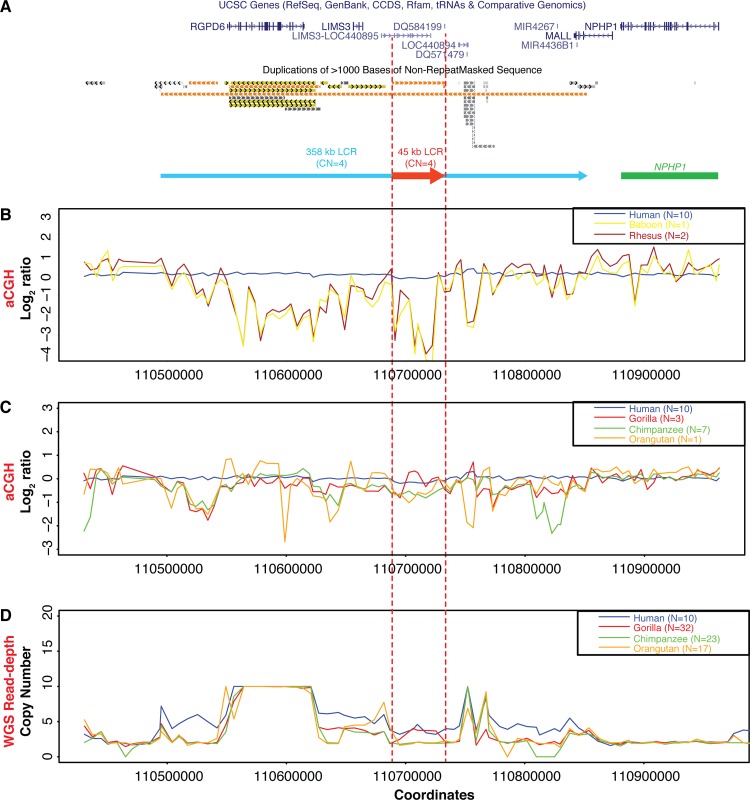
Copy number analysis of genomic sequences flanking *NPHP1* in primates. **A.** UCSC Genome Browser tracks showing the diagram of 358 kb LCRs and 45 kb LCRs on the proximal side of *NPHP1* (Chr2: 110430525–110962639, hg19). Blue and red arrows, one copy of the 358 kb LCRs and 45 kb LCRs for illustration purpose (the copy number of each LCR in NA10851, the universal aCGH reference, is annotated above the arrows); green segment, *NPHP1*. **B-C.** Plot of aCGH species average log_2_ ratios for humans (N = 10), baboon (N = 1), rhesus macaques (N = 2), gorillas (N = 3), chimpanzees (N = 7) and orangutan (N = 1). The probes target the genomic region defined in **Fig 6A**. The species average log_2_ ratio calculated for each probe is used for the plot. **D.** Whole genome sequencing (WGS) read-depth analysis in humans (N = 10), gorillas (N = 32), chimpanzees (N = 23) and orangutans (N = 17). The copy numbers indicated by WGS read-depth data are extracted from the dataset published by Sudmant *et al* [54]. The species average copy number calculated for each position is used for the plot. Blue, humans; yellow, baboon; brown, rhesus macaques; red, gorillas; green, chimpanzees; orange, orangutan. Regions between red dashed lines indicate the region that corresponds to the 45 kb LCRs.

Copy numbers of the 45 kb LCR orthologs in nonhuman primates were also estimated by aCGH using DNA from NA10851 as reference. In the tested baboon (N = 1) and rhesus macaques (N = 2), the species average log_2_ ratios targeting the human 45 kb LCRs were consistent with a complete absence of the 45 kb LCR ortholog (**Fig 6B**). In the tested orangutan (N = 1) and chimpanzees (N = 7), the species average log_2_ ratios indicated a reduced copy number of the 45 kb LCR orthologs in comparison to the reference human DNA used in aCGH (**Fig 6C**). In the tested gorillas (N = 3), the species average log_2_ ratio was close to zero, indicating that the copy number of the human 45 kb LCRs equaled the copy number of the gorilla orthologs (**Fig 6C**). Mean log_2_ ratios of the 45 kb LCRs or orthologous region for each individuals tested, including the hybridizations between human DNA samples without *NPHP1* deletion (N = 10), are shown in **Table 4**.

**Table 4:** Copy number estimation of the human 45 kb LCRs and their orthologs in humans and non-human primates using aCGH.

DNA sample	aCGH mean LR^1^	45 kb LCR CN (estimated)	aCGH DLRS^2^	Species
Baboon1	-1.97	0	0.97	*Papio anubis*
Rhesus1	-1.67	0	1.11	*Macaca mulatta*
Rhesus2	-1.79	0	1.14	*Macaca mulatta*
Orangutan1	-0.54	2	0.54	*Pongo abelii*
Chimp1	-0.66	2	0.31	*Pan troglodytes*
Chimp2	-0.60	2	0.32	*Pan troglodytes*
Chimp3	-0.51	2	0.29	*Pan troglodytes*
Chimp4	-0.67	2	0.28	*Pan troglodytes*
Chimp5	-0.66	2	0.29	*Pan troglodytes*
Chimp6	-0.67	2	0.32	*Pan troglodytes*
Chimp7	-0.67	2	0.33	*Pan troglodytes*
Gorilla1	-0.07	4	0.34	*Gorilla gorilla*
Gorilla2	-0.18	4	0.42	*Gorilla gorilla*
Gorilla3	-0.14	4	0.42	*Gorilla gorilla*
NA18517	0.33	5	0.12	*Homo sapiens*
NA18555	-0.32	3	0.13	*Homo sapiens*
NA15510	0.06	4	0.11	*Homo sapiens*
NA12878	-0.78	2	0.13	*Homo sapiens*
NA07535	0.04	4	0.11	*Homo sapiens*
NA18994	0.04	4	0.12	*Homo sapiens*
NA10860	0.00	4	0.10	*Homo sapiens*
MCF7	-0.30	3	0.12	*Homo sapiens*
H1-P22	-0.18	3	0.14	*Homo sapiens*
H1-P208	-0.22	3	0.10	*Homo sapiens*
PT1^4^	-0.76	2	0.10	*Homo sapiens*, affected
PT2	-0.74	2	0.11	*Homo sapiens*, affected
PT3	-0.79	2	0.11	*Homo sapiens*, affected
PT4	0.02	4	0.11	*Homo sapiens*, affected
PT5	-0.73	2	0.15	*Homo sapiens*, affected
PT6	-0.78	2	0.10	*Homo sapiens*, affected
PT7	-0.65	2	0.31^3^	*Homo sapiens*, affected
PT8	-0.77	2	0.12	*Homo sapiens*, affected

^1^ Mean log_2_ ratio of probes targeting the 45 kb LCRs

^2^ DLRS: Derivative log ratio spread, a measurement of standard deviation of the differences between adjacent points (noisiness) in log ratio data.

^3^ Abnormally high DLRS, may be due to low quality of DNA sample.

^4^ PT1-8: Patients with recurrent homozygous deletion of *NPHP1*.

Similar copy number of LCRs at the human and gorilla *NPHP1* locus could be independently validated using existing and larger datasets. Dumas *et al* performed cDNA aCGH to survey genome-wide gene CNVs across a number of primate lineages [21]. We investigated CNVs at the *NPHP1* locus using their published dataset, and correlated them with the CNV findings from our comparative analysis. Examination of two data points of human cDNAs (*AA937147* and *AI820499*) located at the human 45 kb LCR locus revealed the relative copy numbers examined in each individual comparing to the human reference. Consistently, the gorilla genomes showed roughly comparable signal intensity compared to the human genomes, while the chimpanzee and orangutan genomes presented lower signal intensity for these two data points (**S7 Fig**). Although both *AA937147* and *AI820499* in rhesus macaque and baboon presented lower signal intensity than human, large variations were observed between these two data points (**S7 Fig**). These latter findings in rhesus macaque and baboon suggest that *AA937147* and *AI820499*, although located at the same LCR locus, might have different copy numbers inside each species. Alternatively, the large variation could also be due to the essential absence of the 45 kb LCR ortholog in the macaque and baboon genomes. Moreover, Sudmant *et al* analyzed read-depth profiles from whole genome sequencing (WGS with a median coverage of ~25×) data of 10 humans, 32 gorillas, 23 chimpanzees and 17 orangutans. Copy number analysis based on sequencing read-depth revealed a similar copy number of the 45 kb LCRs and their orthologous regions in the tested humans and gorillas (*p*-value = 0.1705, Welch Two Sample t-test). Moreover, the copy number of the 45 kb LCRs in human is higher when comparing to the orthologous region in the tested chimpanzees (*p*-value < 0.0003, Welch Two Sample t-test) and orangutans (*p*-value < 0.0003, Welch Two Sample t-test) **(Fig 6D** and **S7 Fig**). These aggregated data provided evidence to partially support the observation of the similar genomic architectures between humans and gorillas.

## Discussion

Combined OM and aCGH approaches appear to be a versatile route for delineating SV haplotypes in a structurally complex locus like *NPHP1*. Array CGH can detect CNVs as small as a few hundred base pairs in size in test samples when compared to a reference. However, balanced SVs, e.g. inversions, cannot be detected by aCGH. Unlike aCGH, OM creates large datasets of ordered Rmaps from individual genomic DNA molecules, which through analysis reveal genome structures. Alignments between an optical map from test samples and the *in silico* generated reference maps reveal both CNVs and copy number neutral inversions and translocations. Errors associated with enzymatic cleavage or fragment sizing are inevitable, but can be modeled and dealt with by algorithms and software designed to work with large Rmap datasets for the construction of accurate maps [55–57]. Importantly, the CNVs were called consistently by both OM and aCGH in the samples tested in this study (**Table 2**). These data suggest that OM and aCGH, two orthogonal genomic approaches for SV characterization, can complement each other to provide a comprehensive and accurate SV haplotype. Furthermore, sequencing technologies may greatly facilitate the delineation of SV haplotypes in a region with complex genomic structures. The highly complex and repeating nature of genomic regions enriched with LCRs can challenge short read sequencing approaches and introduce mapping artifacts, some of these limitations may be potentially overcome by single-molecule long-read sequencing technologies, such as single-molecule real-time (SMRT) sequencing [58].

The evolutionary history of structural changes in a complex region such as 2q13 can be reconstructed by appropriate comparisons among related populations and species. The formation of human chromosome 2 through a telomeric fusion of chromosomes 2A and 2B was originally documented using high-resolution G-banding technique [34]. Later, using cosmid sequencing, two inverted arrays of telomeric repeats (5’(TTAGGG)_n_3’) in a head-to-head orientation (5’(TTAGGG)_n_- (CCCTAA)_m_3’) were found at the 2q fusion breakpoint (2qFus) [59]. In our study, we analyzed the origin of the SV haplotypes of the *NPHP1* locus, which is about 3 Mb proximal to 2qFus. The comparative genomic analyses among nonhuman primates and humans suggest a trend of genomic expansion at the *NPHP1* locus during primate evolution. Aggregating the sequence alignments and copy number analyses using aCGH and WGS data of baboon, rhesus macaque, orangutan, gorilla, chimpanzee and human, we propose the following model.

**Fig 7:**
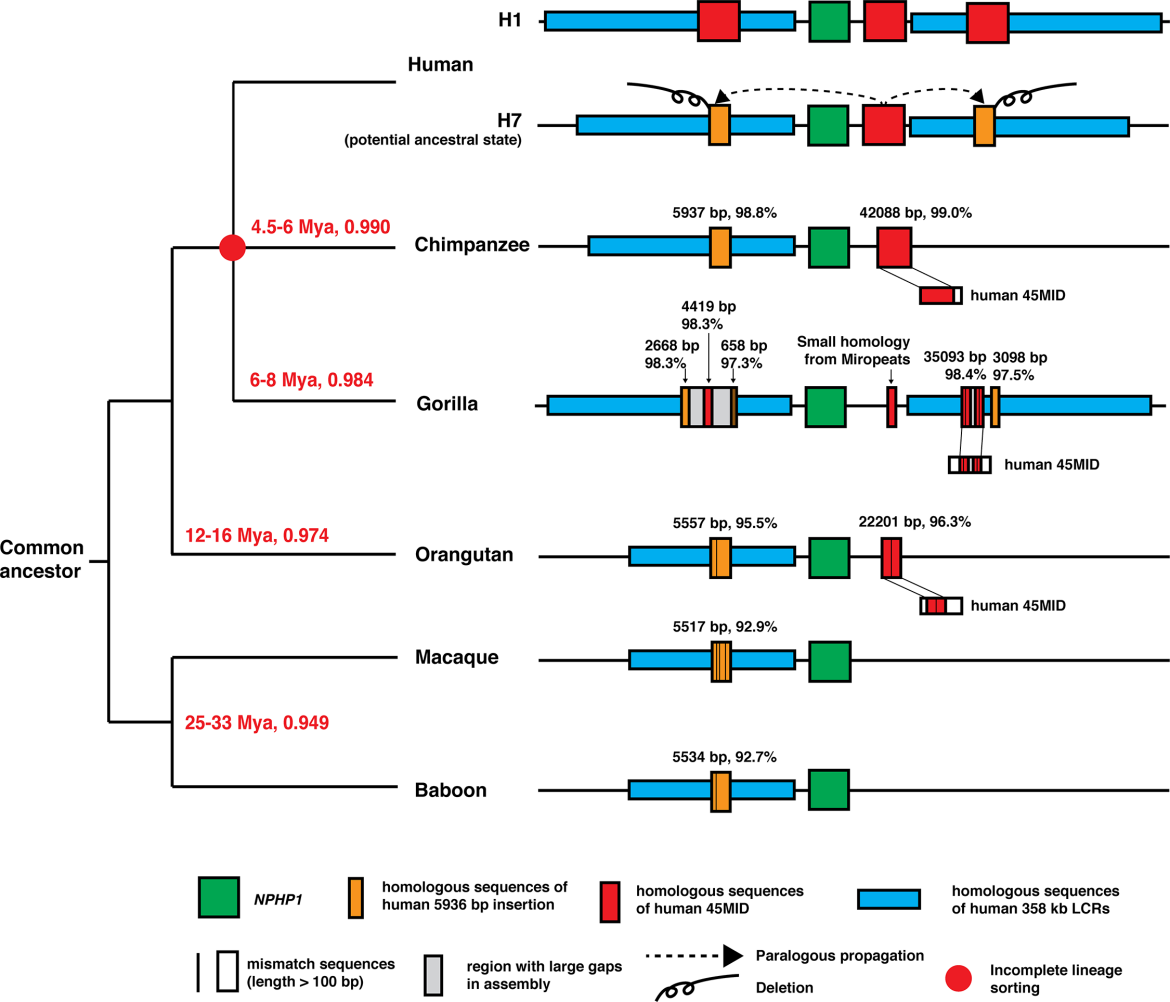
The standard evolutionary phylogeny and a hypothetical model of structural changes from baboon to human. The meaning of each colored boxes or lines are indicated underneath the diagram. The information from **Table 3**, including the BLAT results of human 45MID and 5936Ins in different nonhuman primates, are annotated in each diagram.

First, the present-day human 45 kb and 358 kb LCRs may be formed by gradual expansion and propagation of primate orthologous sequences into paralogous regions early in the evolution of the apes, after they diverged from the Old World monkeys. The nonhuman primate orthologs of the human 45 kb LCRs, not found in the current baboon and rhesus macaque reference sequences, emerge prior to the divergence of orangutan and human as they are found in both lineages (45MID, **Fig 7**). This 45kb sequence expanded in the lineage leading to chimpanzees and exhibit increasing sequence identity to the human 45 kb LCRs. These sequences then propagated into paralogous regions and eventually formed the 45 kb LCRs now observed in the human genome. A similar unknown mechanism may be suitable for explaining the expansion and paralogous propagation of the nonhuman primate orthologs of the human 358 kb LCRs (**Fig 7**). In the human lineage, the order of appearance of the 45 kb LCRs may be inferred by molecular clock analysis based on reference DNA sequence comparisons excluding insertion/deletion (indel) events. Comparative analysis suggests that 45MID in the human haploid reference is the ancestral copy among the three copies. The sequence identity between 45MID and 45PROX or 45DIST is slightly lower than that between 45PROX and 45DIST using the Smith-Waterman local alignment algorithm (**Table 1**). This suggests that 45DIST and 45PROX are paralogous propagation products of 45MID. The observation of the 45MID ortholog location adjacent to *NPHP1* in both chimpanzee and orangutan supports the contention that the 45MID in human is the ancestral copy. However, the sequence-based molecular clock analysis is based on a priori hypothesis that the paralogous LCRs evolved independently. Therefore, it may be confounded by the potential interactions between paralogous sequences, such as homologous recombination leading to gene conversion, considering the fact that the 45PROX and 45MID are imbedded within the two highly homologous and inverted 358 kb LCRs.

Secondly, the human 45 kb LCRs imbedded in the 358 kb LCRs may be formed by paralogous insertion of the duplicated segments at the site where the 5936Ins were lost. This model is supported by the identification of the human 5936Ins orthologous sequences in all the primate reference genomes with increasing identities. The 5936Ins orthologs are present in all lineages studied, whereas the 45 kb LCR orthologs are only present in the orangutan, gorilla and chimpanzee genomes (**Fig 7**). Thus, the haplotype in human with the 5936Ins embedded in the 358 kb LCRs is the likely ancestral haplotype, whereas the haplotype possessing the imbedded 45 kb LCRs in the 358 kb LCRs may be more recent and could be formed by the deletion of the 5936Ins followed by a 45 kb LCR insertion. Therefore, the haplotype without 45PROX and 45DIST may be the most ancestral haplotype in the human lineage.

The structural complexity at the *NPHP1* locus may also exist at a population level within the human species. We delineated various SV haplotypes in seven diploid human genomes and one haploid human genome using OM. These include five diploid genomes (NA10860, NA18994, NA15510, MM52 and HF087) that are homozygous for H2 (H2/H2), one (H1-ES-P208) with a possible admixture of cells with H5/H7 and H6/H7 combinations, one (HCC1937) with H2/H5, and one haploid genome (CHM) with only H5. Within15 chromosomes/SV haplotypes, *NPHP1* inversions are found in at least 11, which account for 73.3% of the total alleles. Moreover, cross-correlation between aCGH (testing copy number of 45 kb LCRs) and OM (testing *NPHP1* inversion) shows that none of the genomes investigated above harbor the haploid reference allele (**Table 2**). On a population basis, 11% of YRI, 3% of CHB/JPT and 1% of CEU harbor 6 copies of the 45 kb LCRs, thus possess at most two reference alleles (homozygous); 31% of YRI, 31% of CHB/JPT and 16% of CEU harbor 5 copies of the 45 kb LCRs, thus possess at most one reference allele (heterozygous) in each examined individual. The aggregate experimental evidence above strongly suggests that the SV haplotype at the *NPHP1* locus is highly variable, as has been observed for the complex LCRs at the iso17q susceptibility locus [33]. Furthermore, the allele represented in the human reference genome may actually be a minor allele, suggesting a potential necessity for improvement of the reference genome and again reiterating the limitations of the haploid human reference and the lack of representation of CNV/SV variant alleles. In the current study, we used OM to identify various SV haplotypes in seven diploid human genomes and one haploid human genome. However, haplotype analysis of the *NPHP1* locus was not performed in a large general population or across ethnicities in either humans or nonhuman primates studied. Thus the frequency of each SV haplotype in the general human population cannot be estimated. Perhaps large-scale assays, with methods such as single-molecule and long-read sequencing, will benefit the further investigation of the complex *NPHP1* locus.

Highly identical LCRs at the *NPHP1* locus lead to local genomic instability, which subsequently results in variable SV haplotypes. A new SV haplotype may be generated from an existing one by a simple inversion between two invertedly oriented LCRs, e.g. H3 from H1 (**Fig 1B**). In some cases, depending on the breakpoint, an inversion may generate a novel SV haplotype that may change the CNV susceptibility of the disease-associated genes, and the resultant SV haplotype may have potential clinical significance [28,29]. At the *NPHP1* locus, the two directly oriented 45 kb LCRs flanking *NPHP1* are the substrates for the NAHR event resulting in the common recurrent deletion of the gene. However, intra-chromosomal NAHR-mediated deletions would be inhibited if a chromosome lacks the flanking directly oriented LCRs. Interestingly, in our study, we identified SV haplotypes that appear to be resistant to NAHR-mediated *NPHP1* deletion as a result of the loss of either one or both of the directly-oriented 45 kb LCRs flanking *NPHP1*. For example, OM analysis of the haploid genome of CHM reveals an H5 SV haplotype losing the 45 kb LCR on the centromeric side of *NPHP1* which is utilized as a flanking substrate for unequal crossing-over (**Fig 3**). Therefore, these findings suggest that there may exist protective alleles that potentially inhibit intra-chromosomal NAHR, while the inter-chromosomal event may also be prevented if the protective allele exists in a homozygous state in an individual. These SV haplotypes could potentially reduce the frequency of the *NPHP1* common recurrent deletion in the human population. This study works in concert with the previous studies regarding the correlation between local genomic structure and individual’s susceptibility to acquiring disease-associated alleles [28,29]. Both protective and susceptible SV haplotypes likely exist at other disease-associated loci with similar structural complexity [22]. The SV haplotypes (H5, H6 and H7) elucidated by OM were identified in human cell lines. Thus, they may potentially reflect tissue culture events generated in the cell lines tested. However, these results are parsimoniously explained by the underlying genomic architecture and mechanistic first principles, and thus the observed results in cell lines likely represent the organismal genome structure, reflect the genomic instability at this locus and indicate the potential existence of H5, H6, and H7 in the personal genomes of individuals in human populations. Nevertheless, it remains to be examined how representative these SV haplotypes are for human populations at large.

The *NPHP1* locus in the gorilla reference genome is an interesting example of evolutionary complexity at two levels: complexity of sequence structure and complexity of population-level evolutionary genetics. Although whole genome comparisons indicate that gorillas diverged from human ancestors before chimpanzees did [60], the gorilla reference genome has a configuration of SV haplotype more similar to human than the chimpanzee does at the *NPHP1* locus (**Fig 7**). Distinctly, instead of completely deleting the gorilla 5936Ins ortholog as observed in the human SV haplotypes, the gorilla 45 kb LCR orthologs appears to be imbedded inside of the gorilla 5936Ins ortholog. The coexistence of these orthologs indicates that the SV haplotype found in the gorilla reference may be an intermediate state different from the SV haplotypes in human. Alternatively, the observation of the human pattern of SV haplotype in gorilla could potentially reflect an assembly error due to the local complexity in the gorilla reference genome; this complexity involves both genomic gaps and the presence of the partial 45 kb LCR and 5936Ins orthologs within the 358 kb LCR orthologs. Analyses using genome-wide cDNA arrays [21], the WGS read-depth analysis, and the genomic aCGH (performed in this study) confirm a comparable copy number of the 45 kb LCR and its ortholog between the human H2 SV haplotype and the gorilla genomes tested (**Fig 6, S7 Fig, S8 Fig, Table 4**). Thus, the aCGH and sequence alignment data are suggestive of an analogous structural haplotype at the *NPHP1* locus between gorilla and human that differs from that of chimpanzee (**S6 Fig**). Copy number analyses of DNA samples from a large number of gorillas will provide additional evidence supporting this hypothesis.

It is intriguing that the gorilla genomic structure for this region appears to be more similar to human than chimpanzee. The estimated divergence time of human-gorilla lineages is approximately 6–8 Mya, which is earlier than estimated human-chimpanzee divergence (approximately 4.5–6 Mya). Moreover, the sequence identity of human versus chimpanzee (99%) is slightly higher than that of human versus gorilla (98.4%) [53,60]. The genome-wide evidence reflects the most commonly accepted evolutionary phylogeny that has humans more closely related to chimpanzees than to gorillas, i.e. ((H-C)-G). However, a recent study of the gorilla genome shed light on the complex evolutionary phylogeny by providing compelling evidence that incomplete lineage sorting affects 15% of the human-chimpanzee-gorilla genomes [60]. That is, whole genome analyses demonstrate that 70% of the gorilla genome follows the ((H-C)-G) standard phylogeny, while 15% of the gorilla genome segments exhibit ((H-G)-C) whereas another 15% exhibit ((C-G)-H). The structural variation results using aCGH and WGS read-depth analysis suggest that the region including *NPHP1* and its adjacent LCRs fall in one of the segments with the alternative ((H-G)-C) pattern. It is plausible that the last common ancestor of humans, chimpanzees and gorillas was polymorphic for *NPHP1* haplotypes, and segregating for haplotypes that resemble the human/gorilla structure and the chimpanzee structure. If this were true, the pattern of variation across the three species can be explained by the retention of one ancestral haplotype in humans and gorillas, and the loss of that haplotype with retention of the more ancestral form in chimpanzees [52,61]. Furthermore, these results lead to the prediction that gorillas will be more susceptible than other nonhuman primates to mutations that delete *NPHP1* and thus cause a disease similar to NPHP1. Our findings also suggest that the complex structure of the human *NPHP1* region was established prior to the fusion of the two ancestral chromosomes that formed the present human chromosome 2, as neither gorillas nor chimpanzees exhibit this fusion.

In summary, we computationally and experimentally characterized the genomic architecture and identified novel SV haplotypes of the *NPHP1* locus in the human 2q13 region. The more commonly observed alternative SV haplotypes suggest the current human genome reference represents a minor allele. For such complex loci enriched with LCRs, the accuracy of assembly may be compromised. Thus detailed exploration using various comparative genomic analytical methods is needed to document the human genome structure and the stages of its evolution in a more comprehensive way. NAHR-mediated *NPHP1* deletion occurs between the two flanking directly oriented LCRs. Here, we found that potential “protective alleles” lacking directly oriented LCR flanking *NPHP1* also exist in the examined human genomes, and such structure may protect those alleles from *NPHP1* deletion mediated by intra-chromosomal NAHR, or inter-chromosomal events in the homozygous state. Such potential “protective alleles” may also exist in other “NAHR-susceptible” loci, with the drawback of such alleles being a decrease in genome plasticity that facilitates evolution. A large number of genomic disorders are associated with loci with complex genomic architectures that introduce risk of genomic rearrangements (e.g. 17p11.2 and SMS/PTLS). The assessment of the disease risk of these disorders can be facilitated by accurate determination of the alternative SV haplotypes via single molecule analysis, including OM or long-molecule/long-read sequencing. Moreover, we assessed the origins of the complexity of the *NPHP1* locus using inter-species comparative genomic analysis, and we found evidence supporting the genomic expansion and propagation of LCRs during primate evolution. The generation of LCRs may occur in a multi-step manner, and the higher order of genomic complexity constituted by LCRs may render the genome susceptible to instability and DNA rearrangements.

## Materials and Methods

### LCR sequence characterizations

Sequences of LCRs were downloaded from UCSC Genome Browser (http://genome.ucsc.edu/cgi-bin/hgTables). The coordinates used for sequence downloading are: chr2:110688766–110733137 (45PROX), chr2:110983705–111031088 (45MID), chr2:111153517–111197896 (45DIST), chr2:110494432–110852754 (358PROX) and chr2:111033788–111392192 (358DIST).

### LCR sequence alignments

Pairwise alignments of the LCRs in the same group were performed using “pairwiseAlignment” R package [62]. It was performed as a type of local alignment that considers the penalty from end gaps. Sequence identity was calculated after the alignment.

### Heat map

We aligned the LCR sequences using the Clustal W2 algorithm [63] and determined the percentage of identical positions over a 100 base pair window along the length of the LCR. We created a position weight matrix (PWM) based on a previously reported recombination hotspot motif [41] and subsequently assessed each LCR for matches to the motif’s PWM and its reverse complement using the Biostrings package implemented in the R Statistical Programing Language (http://www.r-project.org/, http://www.bioconductor.org/packages/release/bioc/html/Biostrings.html). We indicate the positions of strong (>85% of the maximum possible score) matches along the edge of each LCR with a triangle.

### Discordant fosmid selection

Fosmid libraries of 17 individuals (ABC7, ABC8, ABC9, ABC10, ABC11, ABC12, ABC13, ABC14, ABC16, ABC18, ABC21, ABC22, ABC23, ABC24, ABC27, WIBR2, JVI) were downloaded from Human Genome Structural Variation Project (HGSV, http://humanparalogy.gs.washington.edu/structuralvariation/). All end sequence pairs (ESPs) mapped to hg19 build were manually filtered according to the mapping quality and chromosomal location. Discordant fosmids were selected based on the annotations judging the distance and orientation between the ESPs. A UCSC Genome Browser custom track was created for the discordance fosmids identified in *NPHP1* locus based on genomic coordinates and relative orientations of the ESPs (http://genome.ucsc.edu/cgi-bin/hgCustom).

### Miropeats

Miropeats program was used to descriptively illustrate the genomic architecture by plotting the inter-/intra-species alignments of the reference genome. ICAass (v 2.5) algorithm was used to perform DNA sequence comparisons, and Miropeats (v 2.01) was then applied for converting the comparisons into graphical display based on the position and matching quality (a threshold set up by users) [40]. According to the time of divergences and overall sequence similarities upon evolution, different thresholds gauging the length of DNA sequence homology (“seed”) were chosen in order to show the feature of alignments between different primates. In our study, a threshold of 500 were set for baboon/human, macaque/human and orangutan/human pairs, while 1000 were used for gorilla/human, chimpanzee/human and all the intra-species alignments. Miropeats were performed between sequences from genomic intervals +/-800 kb of *NPHP1* of each genome build used.

### Array-comparative genomic hybridization (aCGH)

#### aCGH design

Customized aCGH with high density probes tiling *NPHP1* locus and flanking regions was designed in a 8X60K format using Agilent SureDesign website (Agilent Technologies, https://earray.chem.agilent.com/suredesign/). Reference genome of hg19 assembly was used. Because repetitive sequences are not covered by default, we specified the genomic intervals of LCRs of interest (358 kb and 45 kb LCRs) that need to be covered with high-density probes. In order to generate clean hybridization, only one copy of either 358 kb LCR or 45 kb LCR were tiled. Because of the high sequence identity, the log_2_ ratio can reflect the copy number of the LCRs in the test samples. However, the array itself cannot reveal exactly which LCR(s) is/are lost. An overview of the aCGH design can be visualized in **S2A Fig.**

#### aCGH experimental procedures

Nine hundred nano-grams of genomic DNA from proband (sample to be tested) and an equal amount of DNA sample from a control (NA10851) were used. The experimental procedures, including DNA fragmental and labeling, array hybridization, array washing and scanning and imaging processing, were identical for inter-/intra-species aCGH, following the manufacturer’s protocol (Agilent Oligonucleotide Array-Based CGH for Genomic DNA Analysis version 7.2, Agilent Technologies, Santa Clara, California, USA) with modifications [64]. Human reference sequence hg19 assembly was used to define the genomic coordinated of detected CNVs.

aCGH experiments were performed on DNA samples from human subjects without NPHP1 (N = 10), DNA samples from human subjects affected with NPHP1 (N = 8), and nonhuman primate DNA samples of baboon (N = 1), rhesus macaque (N = 2), orangutan (N = 1), gorilla (N = 3) and chimpanzee (N = 7). Human DNA sample of NA10851 were used as the universal reference for the aCGH experiment. The DNA samples of MM52, HF087, CHM and HCC1937 were not available for the experiment.

### Optical mapping

Optical mapping [46,48,55–57,65–71] is a single-molecule, whole-genome analysis system for the comprehensive discovery and characterization of SVs. Large genomic DNA molecules (from 300 kb to multi-Mbs) were extracted, stretched and immobilized on positively charged glass surfaces via capillary flow within microfluidic devices fabricated using soft lithography [65]. Hydrodynamic forces generated by capillary flow combine with DNA/surface electrostatic interactions to stretch and immobilize very long molecules. DNA molecules, thus presented, were restriction digested (SwaI or BamHI, New England Biolabs), stained with YOYO-1 (an intercalating fluorochrome; Invitrogen) and imaged using a custom-designed, fully-automated, epifluorescence microscopy imaging system [65]. Restriction endonuclease sites undergo double-stranded breakage followed by DNA relaxation at the cut ends, which present as micron-sized gaps along stretched DNA molecules. Acquired images were then automatically analyzed using custom machine vision software [65,66], which yielded large datasets of single molecule ordered Rmaps. Using an iterative assembly process that leverages Bayesian inference approaches and cluster computing [46,55–57,67,68], the Rmaps datasets were then assembled into multimegabase map contigs that were later joined to span entire chromosomes. The iterative assembler clusters single-molecule maps using pairwise alignments to a reference genome, and then assembles these map clusters using a maximum-likelihood Bayesian assembler to generate contigs and consensus restriction maps. The assembled genomes were then viewed within a custom genome visualization environment (Genspect) that allows detailed inspection of the primary data underlying called SVs. Lastly, the assembly/analysis pipeline automatically tabulates a list of SVs that were inspected and manually curated in another custom visualization software (GnomSpace) to characterize all SVs in the analyzed genomes.

### Read-depth analysis

Great ape CNV data derived from whole genome sequencing (WGS) were downloaded as bigBed files from http://eichlerlab.gs.washington.edu/greatape-cnv/tracks/, and bigBedToBed was used to extract annotations within the region of interest. The human genome hg18 coordinates of the CNV annotations were converted to hg19 coordinates using liftOver with default parameters. A UCSC track hub was generated in order to visualize the CNVs lifted over to hg19. The average number of copies for each sample (great apes or human) was calculated for each genomic window of 500 bp in the region of interest.

### Fluorescence *in situ* hybridization

Fosmids clones (G248P81805G1, G248P88963C6, and G248P88660A3) were obtained from BACPAC Resources Center (BPRC) as “stab-cultures”. Clones were cultured in LB medium containing 12.5μg/ml chloramphenicol). Fosmids were extracted from a suspension culture with QIAGEN Plasmid Midi Kit. G248P81805G1 is from *NPHP1*; G248P88963C6 is from the 45 kb LCRs; and G248P88660A3 is from a conserved region ~1.3 Mb proximal to *NPHP1*.

Cultured lymphoblastoid cell lines from the individuals NA15510 (human), CRL-1854 (gorilla), and CRL-1868 (chimpanzee) were harvested using 10ug/ml Colcemid (Roche) for 30 minutes followed by 0.075M (hypotonic) treatment for 10 minutes at 37°C. The cells were then fixed using Carnoy’s fixative (3 methanol: 1glacial acetic acid). The cell pellet obtained from the harvesting was used to prepare the “dropped slides” for FISH.

Directly labeled custom “home-brewed” probes were produced from fosmid clones mentioned above. The home brewing process was performed using Nick Translation Kit (Abbott molecular). Green dUTP, Orange dUTP (herein referred to as red) and Aqua dUTP (Abbott Molecular) were used to label G248P81805G1, G248P88963C6 and G248P88660A3, respectively. Signal validation was also verified by observing the metaphases on an inverse DAPI function.

The slides were aged using 2X SSC at room temperature for 2 minutes followed by sequential dehydration using 70%, 80% and 100% ethanol for 2 minutes each. Metaphases on slides were marked and 5ul of the probe mixture was added. Target DNA and the probes were co-denatured at 75°C for 5 minutes followed by hybridization at 37°C for 16 hours. The slides underwent post wash with 2X SSC at 37C for 2 minutes. The slides were left to air dry after post wash, and 10ul of DAPI-II counterstain (Dako, Agilent Technologies) was added to the slides. Metaphases were viewed using Olympus Florescence microscope (Olympus America) and analyzed using Cytovision software v3.6.

## Supporting Information

S1 Fig Characterization of the features of the LCRs at the *NPHP1* locus. **A.** Dot matrix view of the BLAST alignment. Self-alignment of human reference at the *NPHP1* locus (Chr2:110080914–111762639) is shown. Diagonal lines can infer the orientations of the alignments, which also reveal the LCR composition and relative orientation in this region. The diagrams of H1 are shown along the X and Y-axes. Red and blue lines on top of the dot matrix mark LCRs revealed by the dot matrix. **B.** Pairwise alignments of the 45 kb LCRs and 358 kb LCRs. Heat maps illustrate the pairwise alignments of the 45 kb LCRs and the 358 kb LCRs. Pairwise alignments are performed between each LCR and the consensus sequences of each LCR group. Different colors on the heat map represent a scale of percent sequence identities. Positions of PRDM9 hotspot motifs are marked above each heat map in grey triangles. The color scale in the right bottom corner represents the scale of percent sequence identities.
(TIF)

S2 Fig FISH analysis performed on human, chimpanzee and gorilla lymphoblastoid cell lines. **A.** Above (H1/ref) shows the schematic diagram of the SV haplotype in the reference genome (hg19) at the *NPHP1* locus, with the location of the FISH probes and the distance between different components annotated. Below (H2/NA15510) shows the schematic diagram of the SV haplotype delineated by OM in NA15510 and the FISH probe configuration. **B.** Interphase FISH analysis of NA15510. 42/50 cells scored show four red signals and two green signals (R:G = 2:1). The left three images show green, red and merged signals, respectively. The zoomed-in image of the merged signals is shown on the right. Patterns of red-green-red and yellow-red are shown. The yellow signal is likely to represent overlapping signal of red and green due to their close physical proximity or overlapping signals in the z–plane due to the three dimensional spatial orientation. **C.** Interphase FISH analysis with the “anchor probe” included. **D.** Interphase FISH analysis of CRL-1868 (chimpanzee). A red-green (R:G = 1:1) pattern is observed in 50/50 cells scored. **E.** Interphase FISH analysis of CRL-1854 (gorilla). A red-green (R:G = 1:1) pattern is observed in 46/50 cells scored. Interestingly, despite limited resolution due to close physical proximity, resolved signals of four red and two green (R:G = 2:1) was observed in a minority population (4/50) of gorilla interphase cells, indicating a potential two-copy configuration of the 45 kb LCR ortholog in the gorilla haploid genome. However, this observation could potentially reflect some cells in S or G2 phase of the cell cycle. Dark blue, DAPI counterstaining; light blue signal, the “anchor probe” targeting a conserved region ~1.3 Mb proximal to *NPHP1*; green signal, the probe targeting *NPHP1*, and its orthologs in chimpanzee and gorilla; red signal, the probe targeting the 45 kb LCRs, and its orthologs in chimpanzee and gorilla.
(TIF)

S3 Fig aCGH interrogating the *NPHP1* locus and its flanking sequences in the human 2q13 region. **A.** aCGH design. Probes are shown as black bars. High-density probes are tiled at *NPHP1* locus and its surrounding regions, from chromosome bands 2q12.2 to 2q13. The red box indicates the *NPHP1* locus. **B.** aCGH results for samples with known copy number of the 45 kb LCRs. Six samples (NA18517, NA15510, NA18994, NA10860, NA18555 and NA12878) are used as positive controls. The copy numbers of the 45 kb LCRs in these samples were estimated by Conrad *et al* [2], and the aCGH log_2_ ratio plots of the 45 kb LCR are shown. The theoretical log_2_ ratio and experimental log_2_ ratio are shown on the right of each plot. CN, copy number estimated by Conrad *et al*.
(TIF)

S4 Fig Full sequence of the human 5936Ins. Full inserting sequences of fosmid ABC12-47010700M12 from individual ABC12 are shown in orange, while the flanking sequences are shown in black.
(TIF)

S5 Fig Intra-species Miropeats alignments. Reference sequences of different primates were aligned to themselves using Miropeats to show if there exist any patterns of paralogous LCRs. **A.** Human. **B.** Chimpanzee. **C.** Gorilla. **D.** Orangutan. **E.** Macaque. **F.** Baboon.
(TIF)

S6 Fig Inter-species aCGH and Miropeats alignments. **A.** Examples of inter-species aCGH log_2_ ratio plot comparing each of baboon, macaque, orangutan, gorilla and chimpanzee’s genomic DNA to human’s. The diagram of genomic structure including the LCRs is shown at the bottom, and the shades projected from each LCR indicate the position of each LCR on the aCGH log_2_ ratio plots. **B.** Inter-species Miropeats alignments comparing primates from two adjacent lineages. From top to bottom, Miropeats alignments of baboon/macaque, macaque/orangutan, orangutan/gorilla, gorilla/chimpanzee and chimpanzee/human are shown. The diagram of genomic structure of H1 is shown at the bottom, and the shades projected from each LCR indicate the position of each LCR on the Miropeats diagrams.
(TIF)

S7 Fig cDNA copy number analysis (Dumas et al, 2007) reflecting copy number changes of the 45 kb LCR orthologs across eight primate species. The eight primate species included in the analysis are five humans, three bonobos, four chimpanzees, three gorillas, three orangutans, three gibbons, three macaques and three baboons. Two data points (*AA937147* and *AI820499* representing two human cDNAs) located at the human 45 kb LCR locus are identified and utilized to estimate the copy number of the 45 kb LCR in the corresponding species (Dumas et al, 2007). The distribution of the copy numbers in each species relative to human is illustrated as box plots. Blue, *AA937147*; red, *AI820499*; Y-axis, relative copy number ratio between nonhuman primates and human; X-axis, primate species and number of individuals tested in each species.
(TIF)

S8 Fig Copy number analysis of the *NPHP1* locus in primate species. The heat-maps represent the copy number estimated in windows of 500 bp unmasked sequences in 96 great-ape individuals [54]. UCSC Genome Browser track showing the LCR structure in human is displayed above the heat-maps. The region between the vertical red lines represents the 45 kb LCRs in human.
(TIF)

S1 Table Overall sequence identities between nonhuman primates and human and the hybridization quality of inter-species aCGH. (DOCX)
