# Supplementary figures and images for: Comparative Genomic Analyses of the Human NPHP1 Locus Reveal Complex Genomic Architecture and Its Regional Evolution in Primates
Source: PLoS Genet. 2015 Dec 7;11(12):e1005686. doi: 10.1371/journal.pgen.1005686 (PMC4671654; doi:10.1371/journal.pgen.1005686)

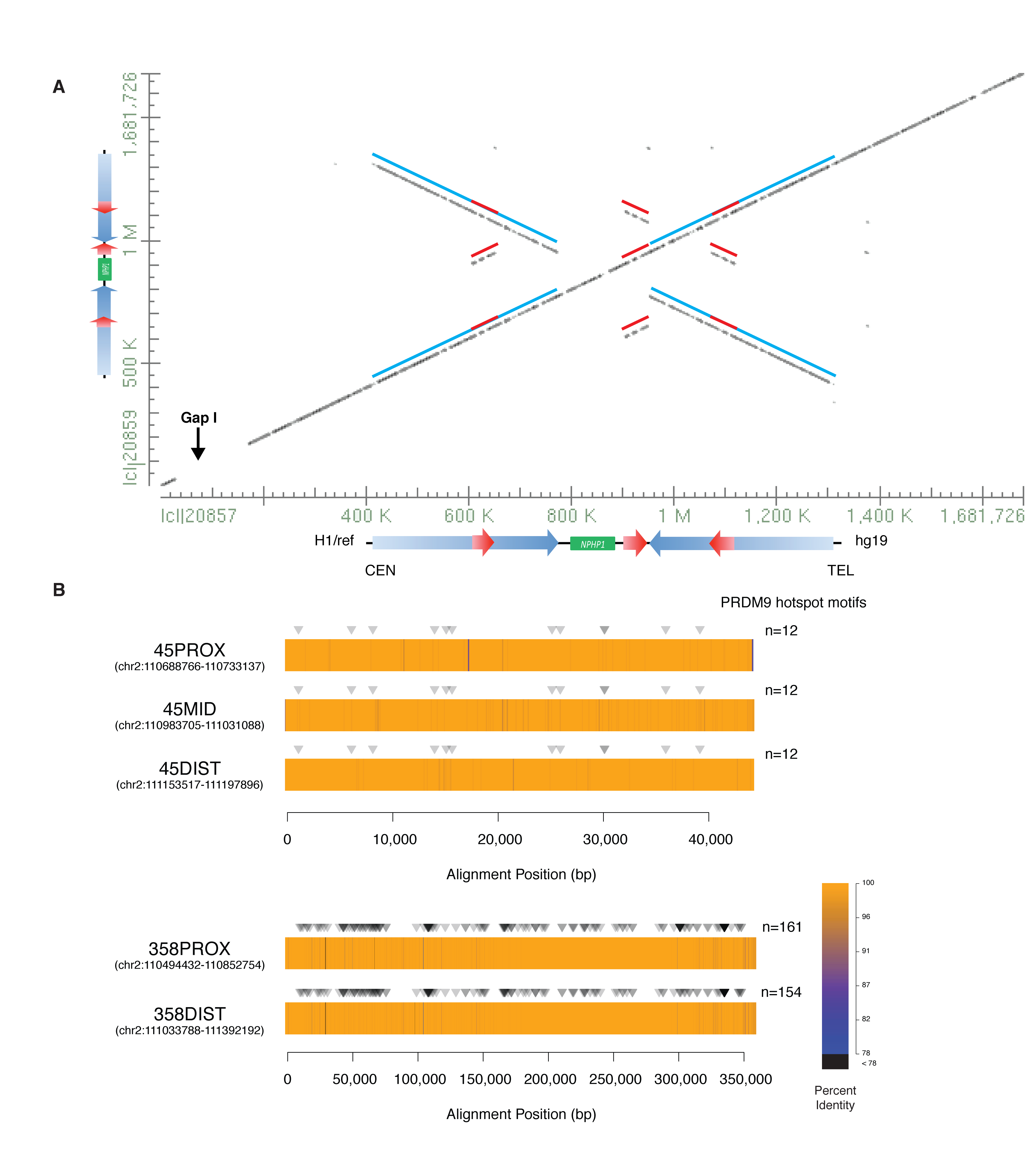

Supplement: S1 Fig — A. Dot matrix view of the BLAST alignment. Self-alignment of human reference at the NPHP1 locus (Chr2:110080914–111762639) is shown. Diagonal lines can infer the orientations of the alignments, which also reveal the LCR composition and relative orientation in this region. The diagrams of H1 are shown along the X and Y-axes. Red and blue lines on top of the dot matrix mark LCRs revealed by the dot matrix. B. Pairwise alignments of the 45 kb LCRs and 358 kb LCRs. Heat maps illustrate the pairwise alignments of the 45 kb LCRs and the 358 kb LCRs. Pairwise alignments are performed between each LCR and the consensus sequences of each LCR group. Different colors on the heat map represent a scale of percent sequence identities. Positions of PRDM9 hotspot motifs are marked above each heat map in grey triangles. The color scale in the right bottom corner represents the scale of percent sequence identities. (TIF) [file pgen.1005686.s001.tif]

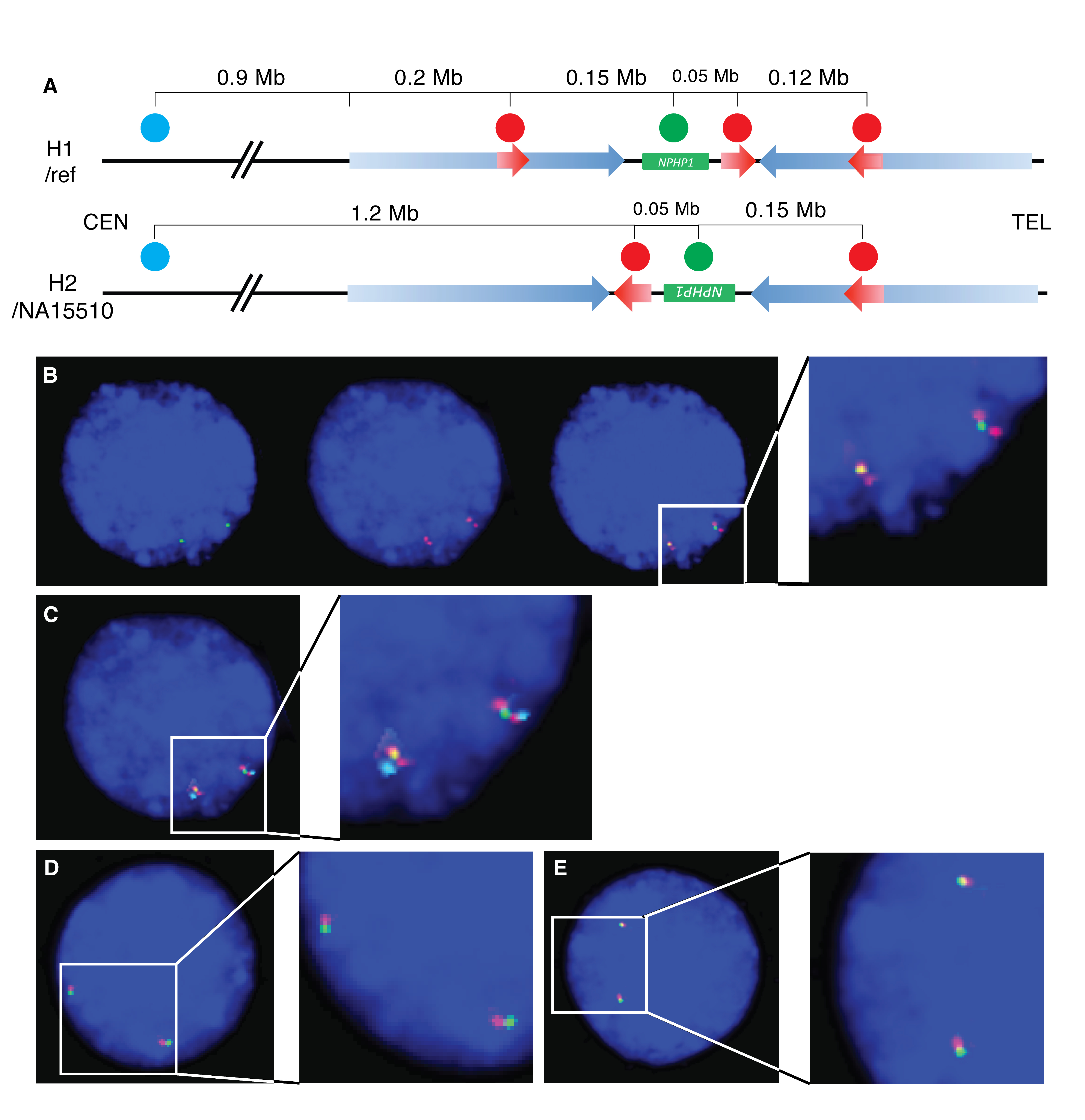

Supplement: S2 Fig — A. Above (H1/ref) shows the schematic diagram of the SV haplotype in the reference genome (hg19) at the NPHP1 locus, with the location of the FISH probes and the distance between different components annotated. Below (H2/NA15510) shows the schematic diagram of the SV haplotype delineated by OM in NA15510 and the FISH probe configuration. B. Interphase FISH analysis of NA15510. 42/50 cells scored show four red signals and two green signals (R:G = 2:1). The left three images show green, red and merged signals, respectively. The zoomed-in image of the merged signals is shown on the right. Patterns of red-green-red and yellow-red are shown. The yellow signal is likely to represent overlapping signal of red and green due to their close physical proximity or overlapping signals in the z–plane due to the three dimensional spatial orientation. C. Interphase FISH analysis with the “anchor probe” included. D. Interphase FISH analysis of CRL-1868 (chimpanzee). A red-green (R:G = 1:1) pattern is observed in 50/50 cells scored. E. Interphase FISH analysis of CRL-1854 (gorilla). A red-green (R:G = 1:1) pattern is observed in 46/50 cells scored. Interestingly, despite limited resolution due to close physical proximity, resolved signals of four red and two green (R:G = 2:1) was observed in a minority population (4/50) of gorilla interphase cells, indicating a potential two-copy configuration of the 45 kb LCR ortholog in the gorilla haploid genome. However, this observation could potentially reflect some cells in S or G2 phase of the cell cycle. Dark blue, DAPI counterstaining; light blue signal, the “anchor probe” targeting a conserved region ~1.3 Mb proximal to NPHP1; green signal, the probe targeting NPHP1, and its orthologs in chimpanzee and gorilla; red signal, the probe targeting the 45 kb LCRs, and its orthologs in chimpanzee and gorilla. (TIF) [file pgen.1005686.s002.tif]

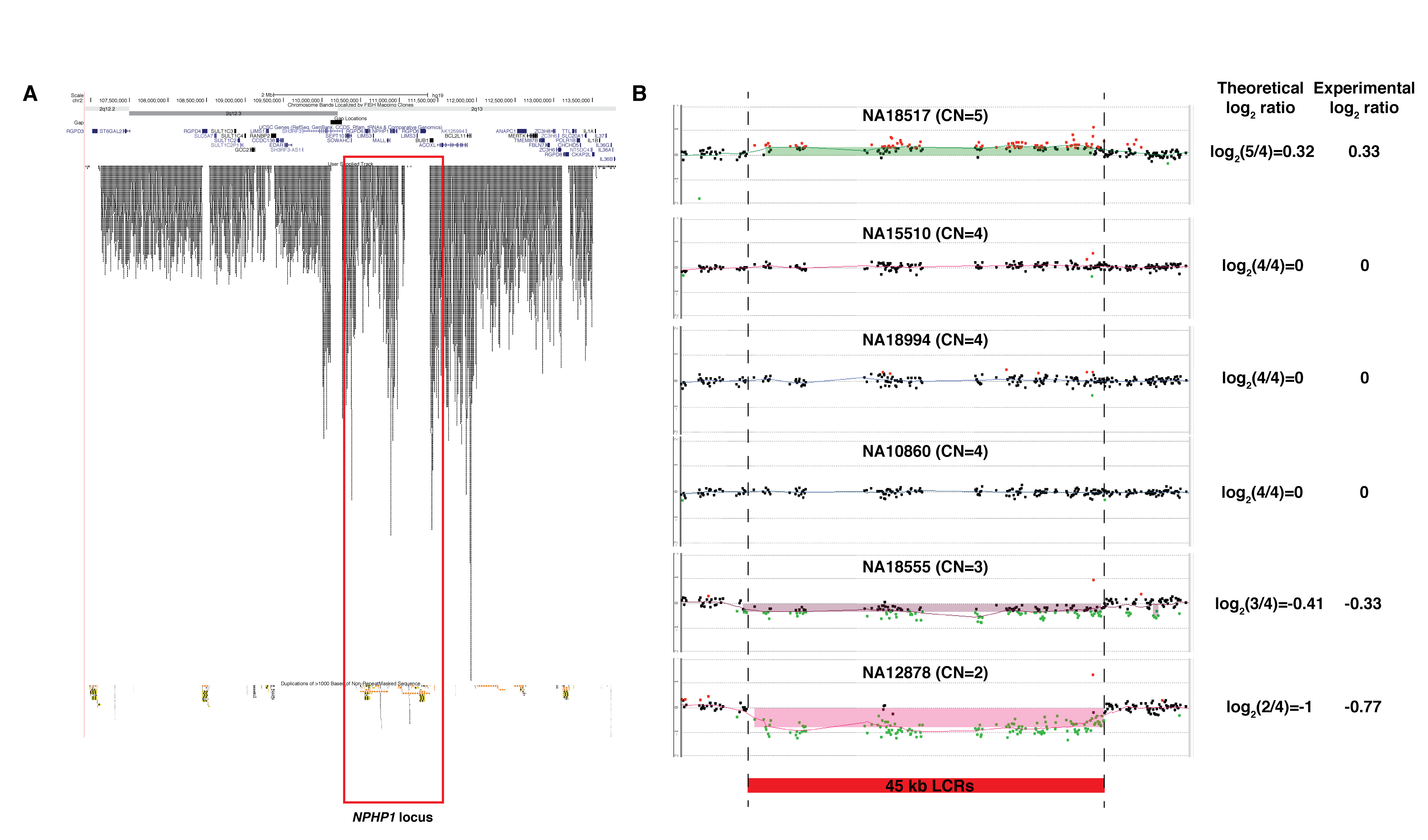

Supplement: S3 Fig — A. aCGH design. Probes are shown as black bars. High-density probes are tiled at NPHP1 locus and its surrounding regions, from chromosome bands 2q12.2 to 2q13. The red box indicates the NPHP1 locus. B. aCGH results for samples with known copy number of the 45 kb LCRs. Six samples (NA18517, NA15510, NA18994, NA10860, NA18555 and NA12878) are used as positive controls. The copy numbers of the 45 kb LCRs in these samples were estimated by Conrad et al [2], and the aCGH log2 ratio plots of the 45 kb LCR are shown. The theoretical log2 ratio and experimental log2 ratio are shown on the right of each plot. CN, copy number estimated by Conrad et al. (TIF) [file pgen.1005686.s003.tif]

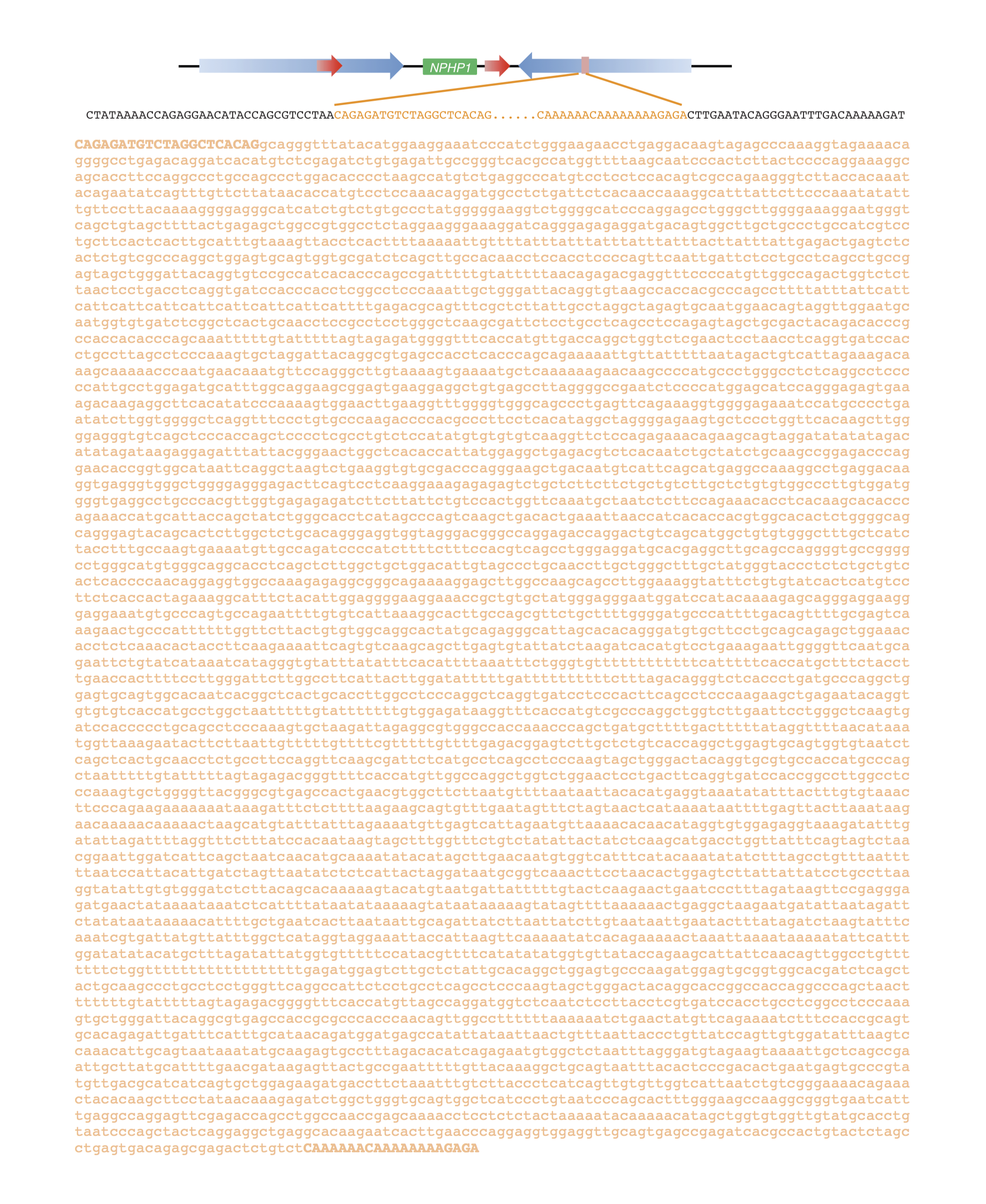

Supplement: S4 Fig — Full inserting sequences of fosmid ABC12-47010700M12 from individual ABC12 are shown in orange, while the flanking sequences are shown in black. (TIF) [file pgen.1005686.s004.tif]

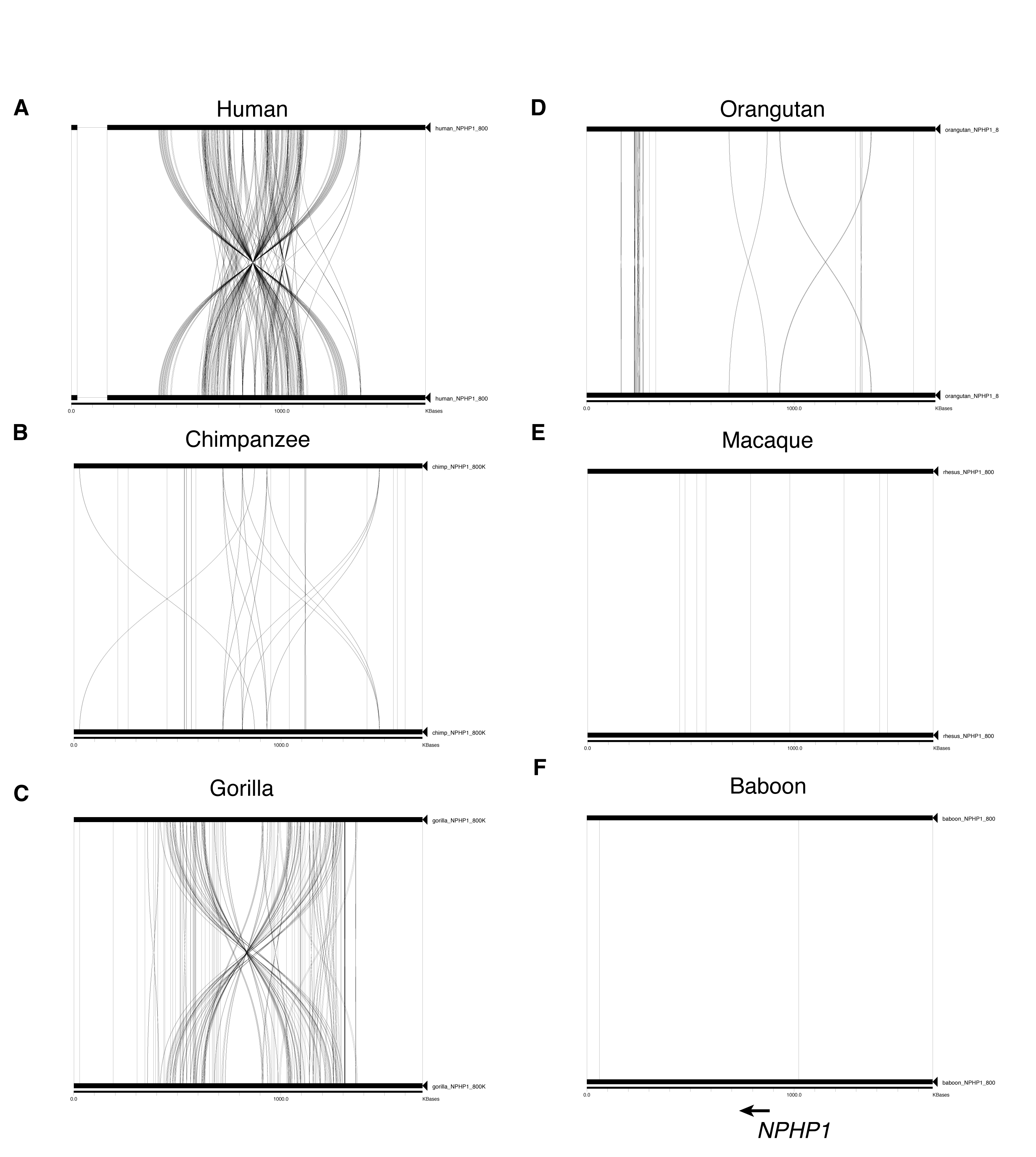

Supplement: S5 Fig — Reference sequences of different primates were aligned to themselves using Miropeats to show if there exist any patterns of paralogous LCRs. A. Human. B. Chimpanzee. C. Gorilla. D. Orangutan. E. Macaque. F. Baboon. (TIF) [file pgen.1005686.s005.tif]

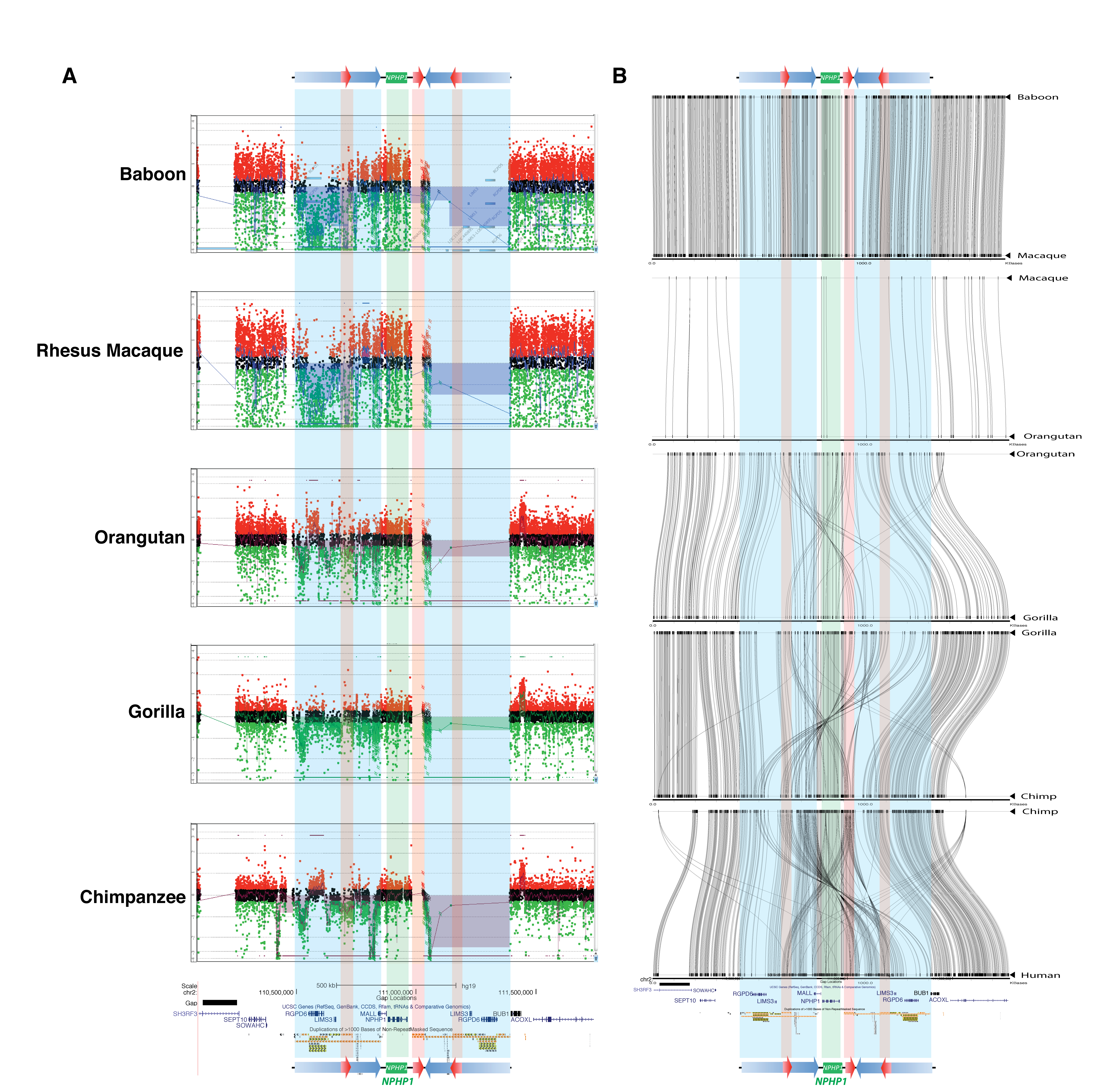

Supplement: S6 Fig — A. Examples of inter-species aCGH log2 ratio plot comparing each of baboon, macaque, orangutan, gorilla and chimpanzee’s genomic DNA to human’s. The diagram of genomic structure including the LCRs is shown at the bottom, and the shades projected from each LCR indicate the position of each LCR on the aCGH log2 ratio plots. B. Inter-species Miropeats alignments comparing primates from two adjacent lineages. From top to bottom, Miropeats alignments of baboon/macaque, macaque/orangutan, orangutan/gorilla, gorilla/chimpanzee and chimpanzee/human are shown. The diagram of genomic structure of H1 is shown at the bottom, and the shades projected from each LCR indicate the position of each LCR on the Miropeats diagrams. (TIF) [file pgen.1005686.s006.tif]

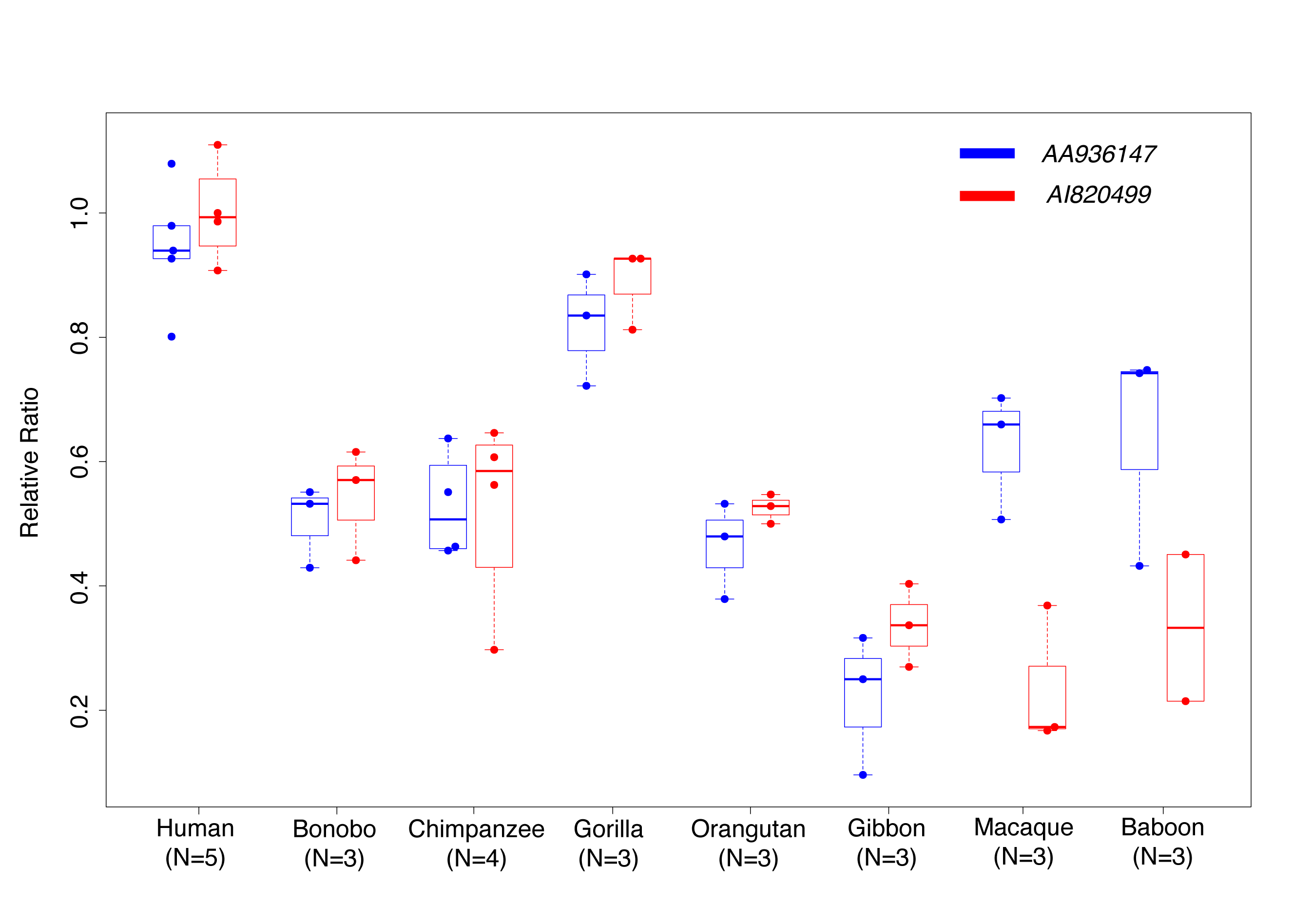

Supplement: S7 Fig — The eight primate species included in the analysis are five humans, three bonobos, four chimpanzees, three gorillas, three orangutans, three gibbons, three macaques and three baboons. Two data points (AA937147 and AI820499 representing two human cDNAs) located at the human 45 kb LCR locus are identified and utilized to estimate the copy number of the 45 kb LCR in the corresponding species (Dumas et al, 2007). The distribution of the copy numbers in each species relative to human is illustrated as box plots. Blue, AA937147; red, AI820499; Y-axis, relative copy number ratio between nonhuman primates and human; X-axis, primate species and number of individuals tested in each species. (TIF) [file pgen.1005686.s007.tif]

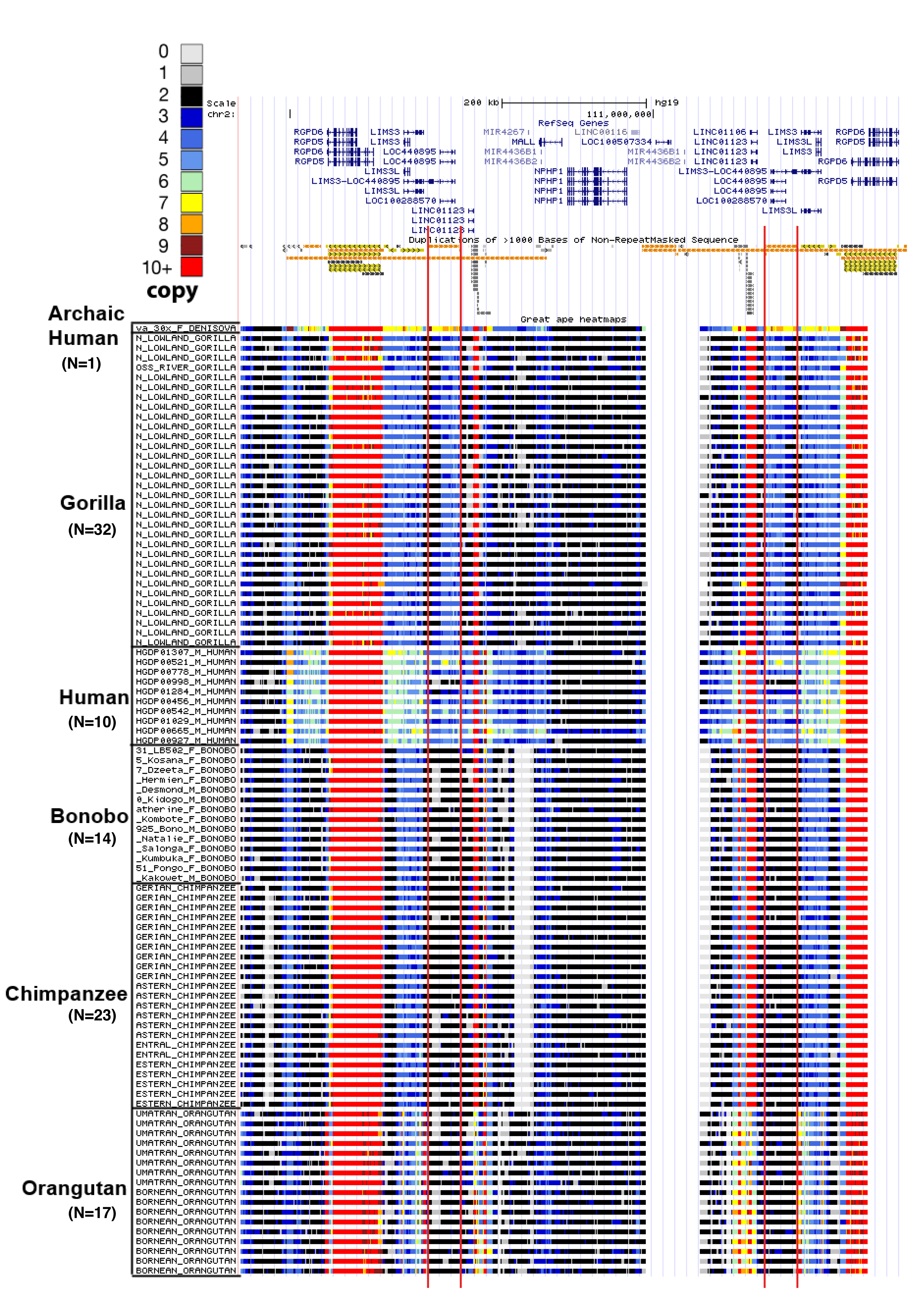

Supplement: S8 Fig — The heat-maps represent the copy number estimated in windows of 500 bp unmasked sequences in 96 great-ape individuals [54]. UCSC Genome Browser track showing the LCR structure in human is displayed above the heat-maps. The region between the vertical red lines represents the 45 kb LCRs in human. (TIF) [file pgen.1005686.s008.tif]
