# Supplementary material for: Comparative Genomic Analyses of the Human NPHP1 Locus Reveal Complex Genomic Architecture and Its Regional Evolution in Primates
Source: PLoS Genet. 2015 Dec 7;11(12):e1005686. doi: 10.1371/journal.pgen.1005686 (PMC4671654; doi:10.1371/journal.pgen.1005686)
Supplement: S1 Table — (DOCX) [file pgen.1005686.s009.docx]

**S1 Table. Overall sequence identities between nonhuman primates and human and the hybridization quality of inter-species aCGH.**

| **Proband**  **(versus human reference)** | Human  (N=10) | Chimpanzee  (N=7) | Gorilla  (N=3) | Orangutan  (N=1) | Macaque  (N=2) | Baboon  (N=1) |
| --- | --- | --- | --- | --- | --- | --- |
| **Sequence identity^1^ (%)** | 100 | 99 | 98.4 | 97.4 | 94.9 | 94.9 |
| **Mean DLRS^1^** | 0.12 | 0.31 | 0.39 | 0.54 | 1.12 | 0.97 |

^1^Sequence identity data are from [Locke et al. 2011](#_ENREF_28) [53].

^2^DLRS: Derivative log ratio spread, a measurement of standard deviation of the differences between adjacent points (noisiness) in log ratio data.
